# In-silico dynamic analysis of cytotoxic drug administration to solid tumours: Effect of binding affinity and vessel permeability

**DOI:** 10.1371/journal.pcbi.1006460

**Published:** 2018-10-08

**Authors:** Vasileios Vavourakis, Triantafyllos Stylianopoulos, Peter A. Wijeratne

**Affiliations:** 1 Department of Medical Physics & Biomedical Engineering, University College London, London, United Kingdom; 2 Department of Mechanical & Manufacturing Engineering, University of Cyprus, Nicosia, Cyprus; 3 Department of Computer Science, University College London, London, United Kingdom; University of California San Diego, UNITED STATES

## Abstract

The delivery of blood-borne therapeutic agents to solid tumours depends on a broad range of biophysical factors. We present a novel multiscale, multiphysics, in-silico modelling framework that encompasses dynamic tumour growth, angiogenesis and drug delivery, and use this model to simulate the intravenous delivery of cytotoxic drugs. The model accounts for chemo-, hapto- and mechanotactic vessel sprouting, extracellular matrix remodelling, mechano-sensitive vascular remodelling and collapse, intra- and extravascular drug transport, and tumour regression as an effect of a cytotoxic cancer drug. The modelling framework is flexible, allowing the drug properties to be specified, which provides realistic predictions of in-vivo vascular development and structure at different tumour stages. The model also enables the effects of neoadjuvant vascular normalisation to be implicitly tested by decreasing vessel wall pore size. We use the model to test the interplay between time of treatment, drug affinity rate and the size of the vessels’ endothelium pores on the delivery and subsequent tumour regression and vessel remodelling. Model predictions confirm that small-molecule drug delivery is dominated by diffusive transport and further predict that the time of treatment is important for low affinity but not high affinity cytotoxic drugs, the size of the vessel wall pores plays an important role in the effect of low affinity but not high affinity drugs, that high affinity cytotoxic drugs remodel the tumour vasculature providing a large window for the normalisation of the vascular architecture, and that the combination of large pores and high affinity enhances cytotoxic drug delivery efficiency. These results have implications for treatment planning and methods to enhance drug delivery, and highlight the importance of in-silico modelling in investigating the optimisation of cancer therapy on a personalised setting.

## Author summary

One of the main challenges in optimising cancer therapy is understanding the in-vivo cancer environment and how it changes over time. The efficacy of chemotherapeutic drugs is known to be strongly dependent on blood vessel wall properties and the architecture of the developing tumour vasculature, which in turn are dependent on biochemical and mechanical interactions between cancer cells and their microenvironment. Here we present a novel in-silico modelling framework of dynamic tumour growth, angiogenesis and drug delivery, and we use it to explore biophysical factors governing the efficient delivery of cytotoxic drugs to solid tumours. We find that the time of treatment and vessel permeability are important factors for the efficacy of chemical agents with low binding affinity, that high affinity drugs can impact the tumour vasculature remodelling and bring vascular structure back to a more normalised state, and that the combination of large-sized vessel wall pores and high affinity enhances cytotoxic drug delivery and efficacy. These results have implications for treatment planning and optimisation, and show how in-silico models can be used to help understand and optimise cancer therapy.

## Introduction

Inefficient delivery of drugs to solid tumours is one of the main reasons for chemotherapy failure. To reach cancer cells, blood borne therapeutic agents have to travel through the tumour vasculature to reach the tumour site, subsequently to extravasate into the tumour interstitial space and finally travel the remaining distance from the blood vessels to cancer cells. Abnormalities in the tumour microenvironment pose major physiological barriers to all three transport steps [1]. The tumour vasculature has an abnormal, chaotic structure with blood vessels being often hyper-permeable, hence, leaving large interendothelial openings, while being tortuous without any particular hierarchy [2]. Furthermore, the structure of the tumour vascular network continuously changes, new vessels are formed owing to hypoxia-induced angiogenesis, while existing vessels might collapse owing to mechanical compression by solid components of the tumour (e.g., cancer and stromal cells and extracellular matrix fibres) [3]. The irregular structure of the tumour vasculature increases geometrical resistance to fluid flow through the vessels thus leading to sluggish blood flow, whereas vessel hyper-permeability might result in excessive plasma (as well as nutrients) loss from the vascular to the interstitial space of the tumour. As a result, these abnormalities can compromise blood perfusion downstream in the vascular network, while vessel collapse might exclude large intratumoural regions from blood supply and, hence, make these regions inaccessible to drugs [2, 4–6]. Therefore, the structure and functionality of the tumour vasculature can determine the amount of drug delivered to the tumour and the efficacy of the therapy [7, 8]. Apart from heterogeneous and low perfusion, other physiological barriers to the delivery of drugs to solid tumours is the uniform elevation of the interstitial fluid pressure owing to: *(i)* the hyper-permeability of the tumour blood vessels, *(ii)* the dysfunction of tumour lymphatics and in some (desmoplastic) tumour types, *(iii)* the dense interstitial space that resist to interstitial fluid flow [9, 10]. All these parameters result in the accumulation of fluid in the tumour and interstitial hypertension, which in term eliminates pressure gradients across the tumour vessel wall and thus, convective transport of drugs [1]. As far as chemotherapy is concerned, cytotoxic drugs have a relatively low molecular weight so that they diffuse fast and do not require pressure gradients for their effective transport [11]. However, binding of the drug to cancer cells might change significantly the penetration and intratumoural distribution of the drug, depending on the binding affinity [12, 13].

To date, mathematical and computational (in-silico) modelling of the tumour–host biophysics and fluid / drug transport phenomena has attracted fair attention in the scientific community. Amongst the early papers in modelling fluid flow in solid tumours—towards studying the interplay between highly permeable walls of neoplastic tumour vessels and interstitial fluid flow—is that of Netti and his colleagues [14]. Their model was capable of predicting the interstitial fluid pressure and velocity profiles, as well as the biomechanics of the tumour and host tissue matrix. Their numerical findings provided valuable insight to understanding the bio-fluid transport in biological tissues, and paved the way for the modelling works that follow. Along the same lines, Pozrikidis and Farrow [15] simulated bio-fluid flow at the interstitium (modelled as an isotropic porous medium) which was described using Darcy’s law, while intravascular flow was described by Poiseuille’s law, and the extravasation using Starling’s law. They investigated fluid flow on an idealised single vessel via a boundary integral formulation to obtain numerically interstitial fluid pressure. Adopting similar governing equations to [15], Soltani and Chen [16] proposed an element-based finite volume methodology to simulate interstitial fluid at the tumour–host tissue. The tumour vascular network was assumed homogeneous, however, their results agreed well with corresponding experimental data of interstitial fluid pressure. Zao et al. [17] developed a computational fluid dynamics animal-specific computational model, informed using dynamic contrast-enhanced magnetic resonance imaging data. The authors investigated the distribution of the interstitial fluid transport within a murine sarcoma with regards to spatially varying properties of the vasculature at the tumour and the host tissue. In 2014, Tang et al. [18] proposed a computational modelling framework of three-dimensional tumour growth and tumour-induced angiogenesis with the aim to evaluate chemical drugs transport. Despite that their model encompassed several bio-physical processes associated with tumour development, such as cell- and vascular-mediated interstitial pressure, angiogenesis, cell proliferation, cytotoxic chemotherapy, etc., the model predictions (tissue growth and vasculogenesis) were not validated against experimental results. Following up [16], Sefidgar et al. [19] investigated numerically, via a model parameter analysis, the effect of tumour shape and size on drug delivery to idealised in shape, solid tumours. The authors concluded that diffusion of cytotoxic drugs is dominant—for most tumour shapes and sizes—while when convection was considerable then drug concentration is larger when compared to similar size tumours. However, their model accounted for several simplifications, such as a homogeneous vascular network, regular tumour shape, and they did not account for the mechano-biology of the tumour–host tissue or the transient effects of the tumour vasculature. More recently, Dey and Sekhar [20] presented a bi-phase mathematical modelling framework—with the solid phase encompassing cell population, the extracellular matrix and the vasculature, while the fluid phase being the interstitial fluid—of solute (macroscopic) transport in soft biological tissues and solid tumours. The authors studied numerically, the impact of the interstitial space hydraulic conductivity, the rate of the solute supply or drainage from the vasculature and lymphs respectively, and the Thiele modulus (see definition of term therein) on the distribution of the interstitial fluid pressure, velocity and concentration in symmetrical tumours. Also, they investigated the role of the Thiele modulus with respect to the delivery of solutes—with emphasis in nutrients transport—and their impact in the tumour development and necrosis formation.

We propose here a novel multiscale, multiphysics in-silico cancer and drug delivery modelling framework that circumvents the simplifications involved in previously-published relevant modelling efforts. We build on our previous in-silico framework that accounts for tumour growth, angiogenesis and vessel compression, oxygen supply, and solid and fluid stress evolution [21]. The model is extended to account for the delivery of drugs and study the effects of the time of chemotherapy administration on intratumoural drug concentration, vessel remodelling, tumour vessels’ permeability and perfusivity, and tumour growth. Particularly, to quantify the structure and hierarchy of the vessels, we employed two geometrical measures that we have previously shown to adequately characterize the tumour vasculature and can be related to drug delivery: *(i)* the maximum distance to the nearest vessels, *δ*_max_, which is a measure of the avascular spaces in the tumour, and *(ii)* a convexity index, λ, which serves as a measure of the three-dimensional structure of the vascular tree [22]. In principle, the parameter *δ*_max_ quantifies the distance of adjacent blood vessels of the micro-vascular tree and it has been observed—by comparing healthy and tumour vascular networks in healthy and cancerous tissue using imaging—that the maximum distance is significantly increased in some solid tumours (e.g., see in-vivo scans in Fig 2 from [23]). However, to simplify the presentation of the in-silico results, we evaluate here the parameter in dimensionless form, ${\bar{\delta }}_{\text{max}}$, i.e. as the fraction of *δ*_max_(*t*) over *δ*_max_(0), with the latter calculated at the initial ‘healthy’ micro-vascular tree. Hence, at time *t* = 0, the parameter is ${\bar{\delta }}_{\text{max}}=1$ (the overbar is omitted in the remainder of the text and in the figures). Also, λ can be interpreted as a parameter that describes the three-dimensional distribution of the vessels and hierarchy. As such, for healthy vasculature it has been observed that λ takes positive values, while in cancerous tissue λ takes negative values [22]. Thus, for the adopted micro-vascular tree at time *t* = 0, the corresponding parameter is calculated λ(0) ≈ 0.5.

In this contribution, we found that time of drug administration—with respect to the size (or ‘stage’ in other words) of the tumour is critical for the outcome of chemotherapy and that the drug can induce changes in the tumour vasculature bringing it to a more normalised state. Interestingly, in-silico model predictions also revealed a strong relation of intratumoural drug concentration to the permeability of the tumour vasculature and the binding properties of the chemotherapeutic agent.

## Materials and methods

The proposed cancer modelling framework is founded in our previous in-silico multiscale model of tumour growth and tumour-induced angiogenesis [21]. Therefore, we have adopted the same notation convention for the presentation of the mathematical model, which has been extended to include drug transport.

### Biofluid flow

#### Vascular flow model

Following [15, 21, 24], blood flow in the microvascular network is assumed axial, steady, laminar and viscous. Thus, the haemodynamics in the individual capillaries can be mathematically described using Poiseuille’s equation that describes the intravascular flow rate via:

$$\begin{array}{c} {\dot{Q}}_{\text{vsc}}=-\;\frac{\pi R^{4}}{8\;\mu_{\text{B}}}\;\frac{\Delta p_{\text{vsc}}}{L_{\text{vsc}}}\;, \end{array}$$ (1)

where *R* is the capillary lumen radius, while *L*_vsc_ and Δ*p*_vsc_ is the length of a capillary segment and the vascular pressure drop (necessitated to drive blood flow) respectively. However, to simplify the modelling of blood flow in the capillaries, without any detriment to the numerical results accuracy or fidelity, the dynamic viscosity of blood, *μ*_B_, is assumed homogeneous and constant in time.

#### Interstitial flow model

Modelling the extravascular space (i.e., the extracellular matrix and the host/tumour cells) as a porous fluid saturated medium, interstitial fluid flow can be described using Darcy’s law [25, 26]. As such, the volumetric flow rate in the extracellular space is given by

$$\begin{array}{c} {\dot{Q}}_{\text{int}}=-\;K_{\text{int}}\;A_{\text{int}}\;\frac{\Delta p_{\text{int}}}{L_{\text{int}}}\;, \end{array}$$ (2)

where *K*_int_ and *L*_int_ is the average hydraulic conductivity and the relative distance between two material points in the interstitium whose (interstitial) fluid pressure difference is denoted by Δ*p*_int_. The interstitium cross-sectional area, *A*_int_, can be expressed with respect to the mean capillary radius and the vascular density, *S*_vsc_, as $A_{\text{int}}=2\pi \bar{R}/S_{\text{vsc}}$ where $\bar{R}$ the average capillary radius in the local neighbourhood of the connective tissues under consideration [21].

#### Transvascular flow model

Fluid transport across the blood vessel endothelial barrier—occurring as a result of filtration—is mathematically described using Starling’s equation

$$\begin{array}{c} {\dot{Q}}_{\text{trv}}=K_{\text{vsc}}\;A_{\text{vsc}}\;(p_{\text{eff}}-p_{\text{int}})\;. \end{array}$$ (3)

Here *K*_vsc_ is the hydraulic conductivity of the endothelial barrier (having dimensions: m Pa^−1^s^-1^), which can be expressed as a function of the size of the fenestrations on the vessel (pores’ average radius), *r*_p_, the fraction of vessel-wall surface occupied by pores, *γ*_p_, the thickness of the vascular wall, *h*, and the plasma dynamic viscosity, *μ*_P_, via: $K_{\text{vsc}}=\gamma_{\text{p}}\;r_{\text{p}}^{2}\;/\;\left(8\;\mu_{\text{P}}\;h\right)$ [27]. Finally, *A*_vsc_ is the surface area of the blood vessel wall and the “effective” pressure is given by: *p*_eff_ = *p*_vsc_ − (*π*_vsc_ − *π*_int_)*σ*_o_, where *σ*_o_ is the average osmotic reflection coefficient of the plasma proteins, *π*_vsc_ is the osmotic pressure of the plasma at the permeable vascular wall, while *π*_int_ is the corresponding osmotic pressure of the interstitial fluid. We account the contribution of the colloid osmotic pressure of plasma and interstitial fluid for a complete modelling description of the micro-circulation system.

#### Lymphatics flow model

Similarly to the Vascular flow model above, we describe the flow rate in the lymphatic vessels using the Hagen–Poiseuille law

$$\begin{array}{c} {\dot{Q}}_{\text{lmp}}=-\;\frac{\pi R^{4}}{8\;\mu_{\text{I}}}\;\frac{\Delta p_{\text{lmp}}}{L_{\text{lmp}}}\;, \end{array}$$ (4)

where *R* here is the mean lumen radius of a lymphatic segment having length equal to *L*_lmp_, while Δ*p*_lmp_ is the pressure difference on that segment. In the present model, the lymphangions pumping properties and the lymphatic valves resistance to flow features are ignored. Nonetheless, a future physiologically realistic model would incorporate these features by following a similar lumped-parameter modelling approach to Bertram et al. [28]. In addition, to simplify the modelling of lymph fluid flow, the viscosity of the interstitial fluid entering the lymphatic vessels, *μ*_I_, is assumed homogeneous and constant in time.

The flow rate Eqs (1)–(4) are coupled to a one-dimensional finite element model for the vascular and interstitial pressures, as in [11, 21]. Thus, a linear system of equations is formed and solved numerically with respect to the unknown nodal (vascular and interstitial) pressures, subject to pressure/flow boundary conditions on the terminal nodal points of the network. The value set for the material parameters of the above equations are provided separately in S1 Table, while the vascular network model parameters are provided in S2 Table.

### Drug delivery

#### Intravascular drug delivery model

The concentration of the solute (drug) in the blood-stream is denoted by *c*_v_; therefore, considering that inside the microvascular network diffusion is negligible then the transport of the drug is dominated by convection [25, 29], and is expressed via the mass balance equation

$$\begin{array}{c} \frac{dc_{\text{v}}}{dt}+v_{\text{vsc}}\;\frac{dc_{\text{v}}}{dL}=0\;, \end{array}$$ (5)

where *dc*_v_/*dL* the drug concentration gradient on a vascular segment. The (axial) blood mean velocity, *v*_vsc_, is computed after solving the equations governing the intra-, trans- and extra-vascular flow at both the vascular network and the interstitial space (as explained in Biofluid flow): $v_{\text{vsc}}={\dot{Q}}_{\text{vsc}}\;/\left(\pi R^{2}\right)$.

The above differential Eq (5) is discretised using one-dimensional linear finite elements, each one representing a blood vessel segment of the vascular network, and to solve the problem numerically the following boundary conditions (BCs) are utilised. At the outlets, which have to be located substantially distant to the main site of the tumour, zero-flux outflow BC is prescribed, i.e.: *dc*_v_/*dL* = 0. At the inlets, Dirichlet BC is imposed to effectively model the (bolus) injection of the drug, thus, described using an exponential decay function of the form: *c*_v_(*t*) = *c*_v-max_ exp[−*t*/*τ*_c_], where *c*_v-max_ is the maximum dose of the cytotoxic drug that has reached the micro-circulation system at the tumour site, and *τ*_c_ the half-time of the chemical agent. Both model parameters can be modified before the in-silico analysis to effectively control the magnitude and time-pattern of the bolus injection.

#### Extravascular drug delivery model

Following the mechanistic compartmental drug delivery model of Thurber and Wittrup [12], we identify three states of the drug, namely the free-drug, *c*_f_, the bound-drug, *c*_b_, and the internalised drug, *c*_i_, concentration respectively. Balance of all three state variables is described through a set of coupled differential equations—where the free-drug governing equation incorporates the advection of the drug at the interstitial space—which reads

$$\begin{array}{c} \frac{dc_{\text{f}}}{dt}+v_{\text{int}}\cdot \frac{\partial c_{\text{f}}}{\partial X}=\frac{\partial }{\partial X}\cdot [D_{c}\;\frac{\partial c_{\text{f}}}{\partial X}]-k_{\text{on}}\;c_{\text{f}}+k_{\text{off}}\;c_{\text{b}}+\Phi_{\text{vsc}}+\Phi_{\text{lmp}}\;,\;\forall \;X\in \Omega , \end{array}$$ (6)

$$\begin{array}{c} \frac{dc_{\text{b}}}{dt}=k_{\text{on}}\;c_{\text{f}}-k_{\text{off}}\;c_{\text{b}}-k_{\text{int}}\;c_{\text{b}}\;,\;\forall \;X\in \Omega , \end{array}$$ (7)

$$\begin{array}{c} \frac{dc_{\text{i}}}{dt}=k_{\text{int}}\;c_{\text{b}}-\delta_{\text{i}}\;c_{\text{i}}\;\epsilon \;,\;\forall \;X\in \Omega , \end{array}$$ (8)

where *k*_on_, *k*_off_ and *k*_int_ are the association (binding), disassociation and internalisation rate coefficients respectively, while *δ*_i_ is the decay rate of the drug—owing to the depletion of the cancer cells after the drug has found its target and the drug natural decay. Since we study here the interaction of the drug with the cancer cells, the above rate parameters take non-zero values in the tumour region, *Ω*^T^, and zero in the host tissue region, *Ω*^H^ (see S3 Table). The free-drug isotropic diffusion coefficient at the interstitium can be obtained using the Stokes-Einstein relationship—valid for when modelling chemotherapy transport [30]:

$$\begin{array}{c} D_{c}=\frac{k_{\text{B}}\;T}{3\;\pi \;\mu_{\text{I}}\;s_{\text{c}}}\;, \end{array}$$ (9)

where *k*_B_ the Boltzmann’s constant, *T* the absolute temperature, and *s*_c_ the size of the molecule (or hydrodynamic diameter if assumed spherical) of the cytotoxic drug. Note that in Eq (6) interstitial flow is assumed incompressible, i.e. (∂/∂**X**) ⋅ ***v***_int_ = 0, where the interstitial fluid velocity can be evaluated using Darcy’s law: ***v***_int_ = −*K*_int_ ∂*p*_int_/∂**X**.

By employing Starling’s equation to relate the rate of solute transport per unit volume through the microvascular endothelial wall into the interstitium [31], one obtains

$$\begin{array}{cc} \Phi_{\text{vsc}} & =P_{\text{vsc},\text{d}}\;S_{\text{vsc}}\;\left(c_{\text{v}}-c_{\text{f}}\right)\\ & +\{\begin{array}{c} \;K_{\text{vsc}}\;S_{\text{vsc}}\;\left(1-\sigma_{\text{f},\text{vsc}}\right)\;\left(p_{\text{eff}}-p_{\text{int}}\right)\;c_{\text{v}}\;\;\;\text{if}\;p_{\text{eff}}>p_{\text{int}}\;\text{else}\\ \;K_{\text{vsc}}\;S_{\text{vsc}}\;\left(1-\sigma_{\text{f},\text{vsc}}\right)\;\left(p_{\text{eff}}-p_{\text{int}}\right)\;c_{\text{f}} \end{array}, \end{array}$$ (10)

where *P*_vsc,d_ is the diffusive permeability of the blood vessels with respect to the solvent (drug molecule), and *σ*_f,vsc_ the solvent drag reflection coefficient at the blood vessel wall. Both latter quantities can be estimated numerically as a function of the drug size to the size of the pores of the vessel wall [27].

Also, *S*_vsc_ is the surface area per unit volume of tissue at the interstitium (otherwise referred as blood vessels’ density).

Similarly, the lymphatic (drainage) contribution in Eq (6) can be expressed as

$$\begin{array}{c} \Phi_{\text{lmp}}=K_{\text{lmp}}\;S_{\text{lmp}}\;\left(1-\sigma_{\text{f},\text{lmp}}\right)\;\left(p_{\text{lmp}}-p_{\text{int}}\right)\;c_{\text{f}}\;, \end{array}$$ (11)

where *K*_lmp_ the hydraulic permeability of the lymphatic wall, and *S*_lmp_ the lymphatic vessels’ density. The solvent drag reflection coefficient at the lymphatic vessel wall, *σ*_f,lmp_, can be also estimated numerically using Deen’s formulas [27]. For simplicity, in the present work, the lymphatic pressure is assumed constant and equal to: *p*_lmp_ = 0 in the entire lymphatic network.

The above coupled reaction-diffusion-convection Eqs (6)–(8) can be solved with the existing 3D finite element framework. The initial condition to this problem is zero drug concentration everywhere in the domain of analysis (i.e., *c*_f_ = *c*_b_ = *c*_i_ = 0), while zero flux boundary condition is applied on the external boundaries of the domain of analysis (i.e. $\partial c_{\text{f}}\left(X,t\right)/\partial \hat{n}=\partial c_{\text{b}}\left(X,t\right)/\partial \hat{n}=\partial c_{\text{i}}\left(X,t\right)/\partial \hat{n}=0$, ∀**X** ∈ Γ). Also, we denote here the total drug concentration that has ‘targeted’ the cancer mass with *c*_h_, which is equal to the sum of the bound-drug concentration in the tumour, *c*_b_, and the internalised (within the tumour cells) drug concentration, *c*_i_. The drug delivery model parameters of Eqs (6)–(8) are provided in detail in S3 Table, while the blood and lymphatic vessels’ hydraulic properties are listed in S1 Table.

### Tissue biomechanics

#### Extracellular matrix structural model

Following Vavourakis et al. [21], structural changes at the extracellular matrix (ECM) of the host and the solid tumour tissue—symbolised in both cases with the state variable *ϵ*—are modelled via a first-order ordinary differential equation:

$$\begin{array}{c} \frac{d\epsilon }{dt}=M-\delta_{\epsilon }\;\mu \;\epsilon -C\;,\;\forall \;X\in \Omega , \end{array}$$ (12)

where *δ*_*ϵ*_ is the degradation rate of the ECM structural components (given in days^-1^) owing to the matrix degrading enzymes (MDEs), secreted by the tumour and the tip-endothelial cells of the tumour vasculature, and the effect of the cytotoxic drugs in depleting the tumour respectively. Variable *μ* denotes the concentration of the MDEs in the matrix, where detailed description of the balance PDE for *μ* can be found in [21] (see Eq (14) therein). The rate-function (in day^-1^) describing the remodelling of the ECM, for both the host and tumour region, is expressed as: $M\left(\xi ,\epsilon \right)=\lambda_{\epsilon }\;\xi \;\text{exp}[-2\;\epsilon /\bar{\epsilon }]$, where $\bar{\epsilon }$ is a scaling parameter that modulates the ECM level at which natural remodelling of the host-tissue matrix occurs, and λ_*ϵ*_ is an ECM remodelling rate parameter. The last right-hand-side term in Eq (12) represents the depletion of the ECM, which is applicable only for the tumour region, *Ω*^T^. This is due to the effect of the cytotoxic agent that has already found its target (cancer cells) in regressing the tumour. As such, the matrix reduction-rate function is expressed by the following polynomial expression

$$\begin{array}{c} C\left(c_{\text{i}},\epsilon \right)=\{\begin{array}{cc} \delta_{\text{d}}\;\epsilon \;{\left(c_{\text{i}}-{\bar{c}}_{\text{i}}\right)}^{a_{\text{d}}} & \text{if}\;\epsilon >{\bar{\epsilon }}_{\text{d}}\\ 0 & \text{elsewhere} \end{array}\;,\;\forall \;X\in \Omega^{\text{T}}\;, \end{array}$$ (13)

where *δ*_d_ is the ECM decay rate (in days^-1^) and ${\bar{\epsilon }}_{\text{d}}$ is a threshold value above which the cancerous ECM is depleted—if only a significant population of cancer cells resides inside the region *Ω*^T^—while ${\bar{c}}_{\text{i}}$ and *a*_d_ are scalar model parameters controlling the reduction-rate function, *C*. The parameter values are provided in part in S3 and S4 Tables, including references to the relevant literature.

#### Tissue solid biomechanics model

Following the continuum mechanics approach of Fung [32], growth of the tumour/host tissue biomechanics is described by introducing an elastic (reversible) part, **F**_e_, and an inelastic (growth) part, **F**_g_, to the deformation gradient tensor: **F** = **F**_e_ ⋅ **F**_g_. As in [33, 34], tumour growth is assumed isotropic via: **F**_g_ = λ_g_ **I**, where **I** the identity tensor. The volumetric stretch ratio λ_g_ is related to the Green-Lagrange volume strain through $\vartheta_{\text{g}}=\left(\lambda_{\text{g}}^{2}-1\right)/2$. However, in the present tumour model, permanent volumetric deformation is expressed explicitly with respect to the oxygen concentration, *ξ*, that promotes cancer cell proliferation, as well as with respect to the tissue structural integrity, *ϵ*, within the tumour (i.e. ∀**X** ∈ Ω^T^). Therefore, for the cancer mass growth, the following modified Gompertz function is proposed

$$\begin{array}{c} \vartheta_{\text{g}}=G\;\;\text{exp}[-\beta_{\text{g}}\;\text{exp}[-\gamma_{\text{g}}\;\xi ]]-G\;\;\text{exp}[-\beta_{\text{g}}]\;, \end{array}$$ (14)

where $G\left(\epsilon \right)=\alpha_{\text{g}}\;\epsilon^{\delta_{\text{g}}}$, and *α*_g_, *β*_g_, *γ*_g_, *δ*_g_ are dimensionless parameters of the growth constitutive function (see S4 Table).

Working in a similar modelling approach to [21], where the MMPs concentration has an effect on the local degradation of the ECM and its impact on the (macroscopic) tissue biomechanics, the modified Neohookean constitutive model has been employed

W¯=m2(I¯1−3)+κ2(J−1)2, (15)

where the structural integrity of the ECM is directly linked to the state variable *ϵ* of Eq (12) via: $m\left(\epsilon \right)=\mu \;\epsilon^{a_{\text{w}}}$ with *a*_w_ being a constant parameter (*a*_w_ > 0) that modulates the tissue softening/stiffening with respect to the ECM density, while model parameters *μ* and *κ* are material constants representing the stiffness of the ECM (equivalent to the shear and bulk modulus respectively for small deformations).

### Angiogenesis and vascular remodelling model

Detailed description of the dynamic angiogenesis model can be found in the recent paper of Vavourakis et al. [21]. In brief, the model is decomposed into to two primary components: *(a)* the model describing the extension of the tip blood vessels (by following a snail-trail modelling approach), the sprouting of blood vessels and the formation of vascular anastomoses, and *(b)* a model that describes the capillary endothelial-wall remodelling and structural integrity with respect to tumour growth-induced solid and fluid mechanical forces. Regarding point *(a)*, vessel-tip and sprout elongation is described via a combination of the chemotactic contribution due to gradients of the angiogenic factors promoting vasculogenesis, the haptotactic contribution due to insoluble gradients of the ECM, and the mechanotactic contribution due to the mechanical forces elevation while the tumour develops in the host. Also, regarding point *(b)*, blood vessel lumen and wall is remodelled with respect to the capillaries haemodynamics. As such, wall shear stress works as a stimulus for vessel remodelling (see Eqs (18)—(20) from [21]), while the balance of fluid pressures (blood pressure and interstitial fluid pressure) and mean solid stresses (tissue hydrostatic pressure) module the state of the vessel (uncompressed, compressed or collapsed; see Eq (21) from [21]). However, in the present in-silico framework, the effect of the drug in vascular remodelling is implicitly accounted for. As explained in the Extracellular matrix structural model sub-section, the cytotoxic agent degrades the tumour following Eq (13), which has a knock-on effect in the volumetric strain, *ϑ*_g_, owing to the tumour development, as well as the tissue macroscopic elastic parameters of the stored-energy function, $\bar{W}$, through the structural integrity / stiffness parameter of the ECM, *m* in Eq (15). This in turn, as demonstrated in the Results and discussion section, is expected to dynamically impact the loading state of the vessels—for example by decompressing due to tumour regression existing mechanically-loaded capillaries. Evidently, vessel decompression improves blood perfusion in the vascular network and, thus, inherently promotes the remodelling of the blood vessel wall and lumen size.

Finally, one modelling aspect the present in-silico framework does not account for is lymph-angiogenesis or the mechanics of lymphatic vessels in response to external mechanical stimuli. However, we leave this as a future modelling development to the present in-silico framework.

### Tissue biochemical model

The governing equations describing the biochemical model of the in-silico framework, i.e. the balance of the oxygen/nutrients, the tumour-angiogenic factors, and the matrix degrading enzymes are defined in detail in our recent paper (see Eqs (12)—(14) in [21]). The adopted material parameters are listed in S3 Table therein, while some few parameters have been updated to reflect the impact of the cytotoxic drug in the extracellular matrix dynamics (see Eq (12)). The latter are listed in S3 Table.

### Solution strategy

**Fig 1:**
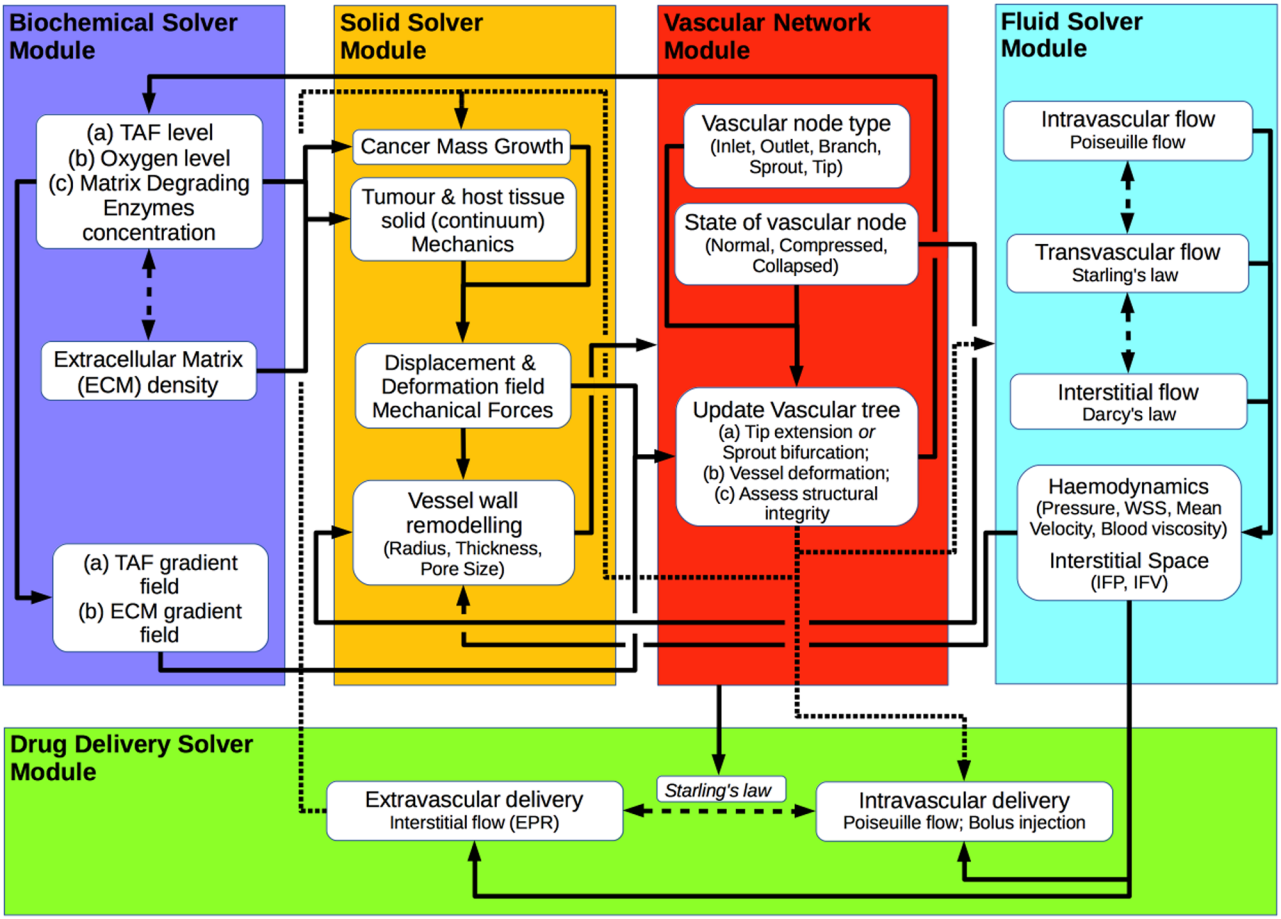
Flow diagram of the coupled in-silico cancer modelling solver. Diagram depicting all major compartments of the multiscale, multiphysics, in-silico cancer, angiogenesis and drug modelling framework, where the interaction between the biochemical and drug delivery module, the vascular network module and the solid and fluid mechanics solver modules is illustrated using arrows. Dotted arrows denote an implicit interplay between the corresponding compartments of the multiphysics in-silico framework.

The present multiscale, multiphysics, in-silico modelling framework consists of five interconnected core compartments that encompass different aspects of the tumour–host micro-environment mechano-biology. The compartments of the framework—called here modules—are the *Vascular Network Module*, the *Biochemical Solver Module*, the *Solid Solver Module*, the *Fluid Solver Module* and the *Drug Delivery Solver Module*. The corresponding modules and building blocks of the proposed in-silico framework are illustrated in Fig 1, which depicts in a flow diagram the interaction among them.

The numerical procedure of the coupled in-silico tumour-growth, hypoxia-induced angiogenesis and drug delivery solvers, in Fig 1, employs four different time discretisation scales, with separate time-step for each of the four solver modules:

1. the time (integration) step of the reaction-diffusion equations of the *Biochemical Solver Module* (see Eq (12) above and Eqs (12)—(14) from [21]), which is of the order of seconds;
2. the time increment between successive solutions of the linear momentum equation for the (tissue mechanics) *Solid Solver Module* (see Eq (2) from [21]), which is of the order of hours;
3. the time increment used to update the tree of the *Vascular Network Module*, which is of the order of hours;
4. the time interval between successive simulations of the *Fluid Solver Module*, which is also of the order of hours (see Eqs (1)–(4)), and
5. the time (integration) step used to solve the drug transport equations of the *Drug Delivery Solver Module* (see Eqs (6)–(8)), which is of the order of a fraction of seconds.

It is important to highlight here that spatial discretisation of the weak-form of the governing equations has been carried out using the Finite Element (FE) method, while time-stepping for the five modules has been implemented in a staggered manner. As illustrated in S1 Fig, the equations involved in the *Biochemical* and *Solid Solver Module* and the extravascular model of the *Drug Delivery Solver Module* were discretised using three-dimensional FEs, while for the intravascular model of the *Drug Delivery Solver Module* and the *Fluid Solver* and the *Vascular Network Module* a one-dimensional FE discretisation has been adopted (for further modelling details read [21]).

Numerical solution of the balance equations has been accomplished sequentially and according to the following order: First, the *Biochemical Solver Module* PDEs are solved together, the solution of which is projected into the *Solid Solver Module* and equilibrium of solid forces is sought numerically (owing to the tumour development). Subsequently, the solution of both above-mentioned modules is transferred into the *Vascular Network Module* and the tree gets updated (i.e., sprouting, branching, vessel compression, etc.). Then, the *Fluid Solver Module* is invoked to compute the interstitial and intra-/trans-vascular flow. The *Fluid Solver Module* operates at the end of every successful *Solid Solver* and *Vascular Network Module*, and before the *Drug Delivery Solver Module*, due to both blood/plasma and interstitial fluid flow being assumed viscous-dominated (at extremely low Reynolds numbers) and quasi-steady. This is necessary to account: *(a)* for the localised effects of solid stresses elevation or reduction, which is owed respectively to the natural tumour growth or the regression of the tumour in response to the cytotoxic drug, and *(b)* the dynamic changes of the vascular network growth (sprouting, anastomosis) which may directly affect blood flow and, thus, implicitly interstitial fluid flow. With regard to *(a)*, as elaborated in [21], during the course of a simulation solid stresses may increase or decrease which in turn are expected to compress or decompress the capillaries adjacent to the tumour, respectively. This effect, in principle, is expected to modulate directly blood perfusion in the vascular tree and, thus, vascular remodelling in the *Vascular Network Module*. The solution of the *Fluid Solver Module* together with the solution of the *Biochemical Solver Module* is transferred to the *Drug Delivery Solver Module* to compute numerically the drug distribution in the vascular and extravascular spaces. Finally, the state of the vascular segments inside or proximal to the tumour region is re-assessed, and the list of collapsed blood vessels is revised while the microvascular pressure and interstitial fluid pressure distribution is updated by re-invoking the *Fluid Solver Module*. In summary, the coupled solver (that encapsulates all five modules) iterates until the simulation reaches the desired time point (e.g. here is set to 40 days), where the frequency with which each module is invoked depending on the time-step/increment chosen.

The above coupled multiscale numerical procedure is repeated until the termination of tumour growth/angiogenesis/drug transport simulations. Details about the FE implementation of the proposed tumour-induced angiogenesis and growth model are provided in S1 File. The *C++* code of the in-silico cancer modelling platform can be accessed online via Bitbucket from: Finite Element Bioengineering in 3D (*FEB3*).

## Results and discussion

The in-silico model was specified to simulate solid tumour growth, vasculogenesis and subsequent cytotoxic drug delivery in immunodeficient mouse models. We focus on neoplasia in dense fibrous tissue (i.e. high collagen content), and thus mimic desmoplastic tumours (e.g. breast and colorectal cancer). All model/material parameters are presented in the supplementary tables: S1 and S4 Tables. The majority of the parameters were taken from relevant literature, and where possible from published experimental data for MCaIV murine mammary adenocarcinomas.

Simulations were performed for a spherical cancer mass with 1 mm initial diameter inside a ≈ 1.7 cm^3^ cubic domain of the tumour–host tissue. The domain is represented by a three-dimensional finite element mesh, constructed using Gmsh (http://gmsh.info/) and consists of 3,320 hexahedra and 3,963 nodes. Embedded to the three-dimensional (tissue) mesh is a one-dimensional, non-conforming mesh to represent the vascular network, consisting of 2,880 linear line elements (baseline mesh at day 0). S1 Fig depicts the initial three-dimensional mesh of discretised tissue domain and the initial discrete network of the capillaries. The network used here is randomly generated from a uniform distribution, but can be tailored to any physiology.

As explained in the Introduction, we use the variables *δ*_max_ and λ to describe the three-dimensional architecture of the vascular network [22]; that is to say, the collective distribution and arrangement of blood vessels outside the tumour. Specifically, for the *δ*_max_ variable we consider its dimensionless form, i.e. the ratio of the variable at a specific time point with respect to the baseline value (control) at day 0, which designates the transition from avascular to vascularised tumour growth. We will also use henceforth the term ‘vascular architecture normalisation’ to refer to the process of remodelling *δ*_max_ and λ to their normal physiological values [22], while the term ‘vascular normalisation’ is used here to describe the normalisation of vessel wall structure [23]. Hence, we assume here normal physiological values for *δ*_max_ in the value range between 1.0 and 1.4, while for the second parameter when λ > 0.

To test the effect of neoadjuvant vascular normalisation by a vascular normalising agent, we conducted simulations with three different initial values of the size of the vascular wall pores, *r*_p_ (referred hereafter as ‘poresize’ for brevity), ranging from 10 nm to *r*_p_ = 150 nm (see S2 Table). However, the present in-silico framework does not explicitly model for anti-angiogenenic treatment, or the effect of specific vascular remodelling growth factors (e.g., VEGF, PDGF) [35, 36]. As such, and in contrast to for example [37], this model does not account for the effects of VEGF on the permeability of the tumour blood vessels; hence, the values set for the size of the pores were selected to model either no vascular wall maturation / hyperpermeable vessels (*r*_p_ = 150 nm) versus mature / less permeable vessels (*r*_p_ = 10 nm). Also, to simplify the presentation of the in-silico results and aid the discussion, we choose henceforth the two extreme values of *r*_p_, which were termed low and high poresize, respectively. As such, low poresize reflects the case where vascular normalisation has been performed in accordance to previous studies [38].

To test the effect of cytotoxic drug binding properties we also conducted simulations with different rates of affinity, *k*_on_, spanning from 0.005 s^-1^ to 5 s^-1^ (see S3 Table), with the two extremes termed henceforth low and high affinity, respectively. The drug affinity and vessel poresize are kept constant throughout each simulation. Here we focus on cytotoxic drugs, such as paclitaxel, and hence choose a small particle diameter, *s*_c_, which is kept constant for all simulations: 1 nm. The study of larger drug sizes (e.g., liposomes, micella or drug-borne nanoparticles) is reserved for future work.

Finally, we define three tumour stages: early- (10 days from baseline), mid- (20 days from baseline) and late-stage (30 days from baseline). This allows us to test the effect of initial vascular architecture and tumour size on drug efficacy.

### Diffusion transport is the dominant mode for cytotoxic drugs

**Fig 2:**
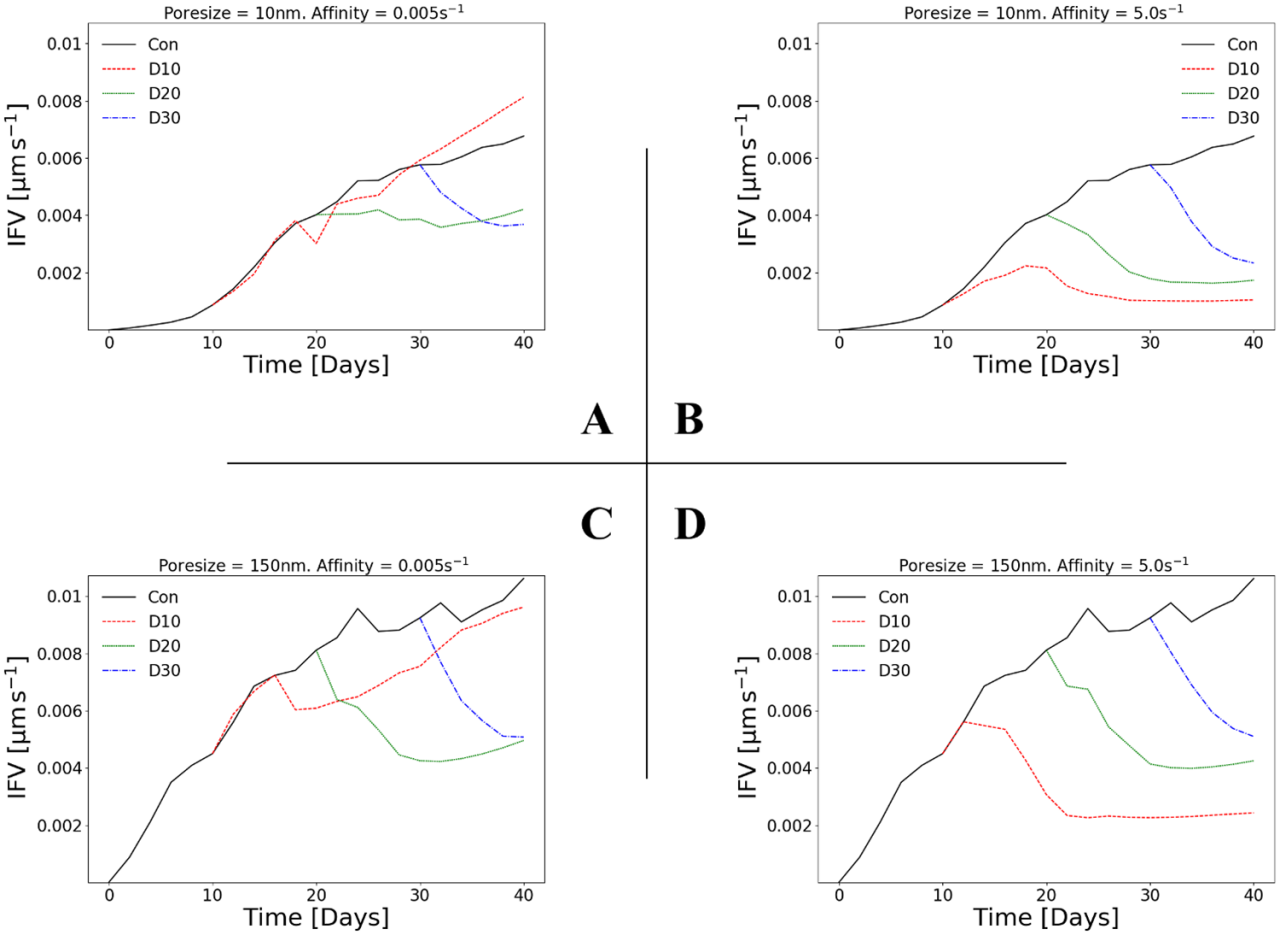
IFV depends on both vessel poresize and drug affinity. Line plots of the averaged interstitial fluid velocity (IFV) magnitude as a function of time. The 2×2 matrix of plots shows the results for two poresizes (A,B: *r*_p_ = 10 nm; C,D: *r*_p_ = 150 nm) and two affinities (A,C: *k*_on_ = 0.005 s^-1^; B,D: *k*_on_ = 5 s^-1^). Each line plot depicts the control (no drug injected), and the in-silico predictions with drug injected at day 10 (D10), day 20 (D20) or day 30 (D30). See also S2 Fig. for simulation results involving intermediate values of poresize and affinity.

For our drug of choice, the relative roles of diffusive and convective transport can be determined by estimating the Péclet number, defined as the ratio between the diffusive and convective flux (see for example [39]). For a molecule of diameter *s*_c_ = 1 nm approximately and the specific interstitium properties (see material parameters in S1 Table), the diffusion coefficient of the drug, *D*_c_, (see Eq (9)) is of the order of 10^−4^ mm^2^s^−1^ approximately. Fig 2 illustrates the averaged interstitial fluid velocity (IFV) magnitude—computed over the tumour and the surrounding host tissue 3D domain of analysis—with respect to time for four cases: low poresize and low affinity (A), low poresize and high affinity (B), high poresize and low affinity (C), as well as high poresize and high affinity (D). The largest IFV is predicted for high poresizes, which is expected—higher poresize means a larger transmural flux of biofluids—with a maximal value ranging between 0.9—1.2 μm s^−1^ approximately. The corresponding Péclet number we evaluate is less than 0.1, which places the mode of transport firmly within the diffusion domain (see also Box 1 in [39]). Therefore, supported by relevant experimental and theoretical observations [25, 26, 40, 41], we confirm that convection has a negligible role in the transport of cytotoxic drugs to the tumour and, hence, the main effect of increasing the poresize is that it also enhances transvascular solute transport (i.e. *Φ*_vsc_ in Eq (6)) and thus the total volume of free drug. This prediction is important when appraising the following results, particularly the effect of poresize and its dependency on drug affinity.

Fig 2 shows that the temporal dynamics of the IFV magnitude (averaged over the tumour and the peri-tumoural tissue area) are also dependent on the injection time and drug affinity. Broadly speaking, this is due to the relationship between tumour volume and IFV. In agreement with [34], as the tumour grows it deforms the surrounding tissue, which elevates the hydraulic conductivity of the tissue at the peri-tumoural stroma and, thus, resulting the IFV to increase (see the control lines in each plot). Conversely, as the tumour is regressed by the drug, the surrounding tissue becomes less deformed and, hence, the IFV decreases. At high affinity (second column in Fig 2), this effect is consistent across all injection times. At low affinity, however, the relationship is more complex: later injections (day 20, day 30) are dependent on both poresize and affinity, with low poresizes causing the IFV to continue to increase or remain constant after injection, respectively.

Comparing quantitatively the results between Fig 2B and 2D, IFV is scaled up by a factor of 2 whereas the hydraulic conductivity of the tumour vessels scales up by a factor of 200 approximately (see *K*_vsc_ definition in Transvascular flow model). Also, in view of Fig 2C and 2D, we can project the efficacy of less diffusive drugs (e.g. nanoparticles), post to administering a cytotoxic agent, be improved for hyperpermeable tumour vessels at early- to mid-stages of the tumour development if only the binding rate of the agent is very low. If the opposite occurs, then follow-up administration of drug-borne vesicles can potentially benefit from the enhanced convection transport for late-stages of the growing tumour and its increased vascular density.

**Fig 3:**
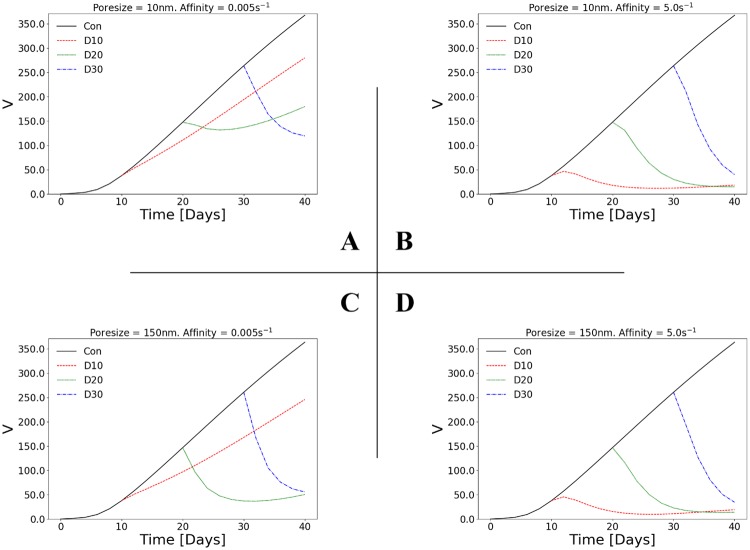
Tumour regression depends on vessel poresize for low affinity but not high affinity drugs. Line plots of the relative tumour volume (*V* = Vol.(*t*)/Vol.(*t* = 0)−1) versus time. The 2×2 matrix of plots shows the results for two poresizes (A,B: *r*_p_ = 10 nm; C,D: *r*_p_ = 150 nm) and two affinities (A,C: *k*_on_ = 0.005 s^-1^; B,D: *k*_on_ = 5 s^-1^). Each line plot depicts the control (no drug injected), and the in-silico predictions with drug injected at day 10 (D10), day 20 (D20) or day 30 (D30). See also S4 Fig for simulation results involving intermediate values of poresize and affinity.

In summary, these predictions reflect the complex relationship between tumour growth and vessel poresize at low affinity, shown in Fig 3, and discussed in detail in the following subsection.

### Vessel poresize affects tumour regression for low affinity but not high affinity cytotoxic drugs: Implications for staged delivery

To investigate the relative effects of drug affinity (binding rate), *k*_on_, and vessel poresize, *r*_p_, on tumour regression, Fig 3 shows the tumour volume over time for four cases: low poresize and low affinity (A), low poresize and high affinity (B), high poresize and low affinity (C), and high poresize and high affinity (D). Each plot shows the control (i.e. no drug injected) and the treated in-silico predictions with injections occurring at three different ‘stages’ with respect to the tumour growth baseline simulations, specifically at day 10, day 20 or day 30 respectively.

The control case shows that after 10 days the tumour grows approximately with constant rate (see also document results of the tumour volume rate in S5 Table), as expected from the Gompertz growth function prescribing the growth. As seen in all plates of Fig 3, the tumour size in the control simulations does not exceed 10 millimetres in diameter, which agrees with the experimentally measured tumour size in the murine models used for this study (e.g., see reported data in [42]). However, the present model can readily be used to simulate large tumour development (e.g. by allowing the simulation to run over longer time periods), as well as other types of solid tumours (e.g. brain gliomas, pancreative tumours, etc.). This could be achieved by adjusting the corresponding in-silico model parameters, as summarised in S1–S4 Tables.

At high affinity (Fig 3B and 3D) the response of the tumour to the drug is consistent across all injection times: the tumour regresses to a minimal value, with volumes from all three injection times converging to approximately the same result by the end of the experiment. Almost no dependency on poresize is observed from the simulation results, which supports the argument about the competition between drug diffusion and its binding rate (see for example Eq (1) in [13]. The above can result from the combination of two factors: *(i)* that diffusion is the dominant mode of transport for chemotherapeutic agents, and *(ii)* that for high-binding drugs the penetration length is small; while for high-binding drugs pertaining very fast diffusion rates, the drug clears out of the tumour quickly. Therefore, the limiting factor can be implicitly regarded as the size of the tumour, since it defines the limit on the mass of internalised drug. Interestingly, at low affinity a dependency on poresize is predicted: in Fig 3A at day 20, the tumour is regressed slightly before relapsing shortly after injection, while in Fig 3C at day 20 the tumour is regressed more before relapsing near the end of the simulation. Similarly, different final values are predicted between low and high poresizes for injections at day 10 and day 30. This suggests that at low affinity a higher volume of drug is necessary to overcome the additional limiting factor of the drug’s slow binding rate, i.e. to increase *c*_b_ such that the tumour size—and hence the mass of drug that can be internalised—becomes the limiting factor. This effect can be predicted in the concentrations of bound/associated and internalised drug, *c*_b_ and *c*_i_ respectively, over time (see S3 Fig): there is a limit beyond which increasing *c*_b_ has little effect on *c*_i_ level.

### Time of drug administration affects tumour regression for low affinity but not high affinity cytotoxic drugs

Another interesting prediction from Fig 3 is that the final tumour volume is independent of the time of injection for high affinity but not low affinity drugs. This suggests that the time of drug administration might be of less importance for high affinity drugs; the tumour is regressed to the same final volume irrespective of its size when the drug is administered. Conversely, the time of injection is crucial for low affinity drugs, with early-stage (day 10) injections only reducing the tumour’s growth rate as opposed to regressing it. Furthermore, we observe for the same drug properties that mid-stage injection are very likely to permit the tumour to relapse (high probability for poorly permeable tumour vessels). On the contrary, late-stage injections (day 30) lead to significant cancer volume reduction, with the rate varying depending proportionally on the permeability of the nascent vessels. S5 Table presents in tabular form the tumour volume rates (in mm^3^ day^-1^) for the control versus the treated simulations (injection times: D10, D20, D30) and for the four combinations of poresize and drug affinity values. In accordance with the results shown in Fig 3, we note that tumour can be regressed significantly up to a ≈ 16 mm^3^ day^-1^ rate for late-stage injections of a high affinity drug; whereas for early-stage injections of the same drug can fairly regress a “premature” tumour at a rate 0.2—0.7 mm^3^ day^-1^ initially, but as depicted in Fig 3B and 3D, the tumour shows a trend towards gradual relapse (after day 30). Contrary to the latter case, early-stage injections of a low affinity drug has negligible impact to the tumour regression. In fact for both low and high poresize (note that the drug size, *s*_c_, is comparably smaller to the poresize, *r*_p_, in both cases) the average tumour development rate—within a 5-day time frame post injection at day 10—is 3.6 and 2.8 mm^3^ day^-1^ respectively, while the corresponding tumour growth rates of the control—at the same time frame—are 5.3 and 5.1 mm^3^ day^-1^ respectively. In summary, the above findings elucidate the implications for the outcome of low/high affinity cytotoxic drugs and the importance of the time of injection with respect to the tumour stage.

**Fig 4:**
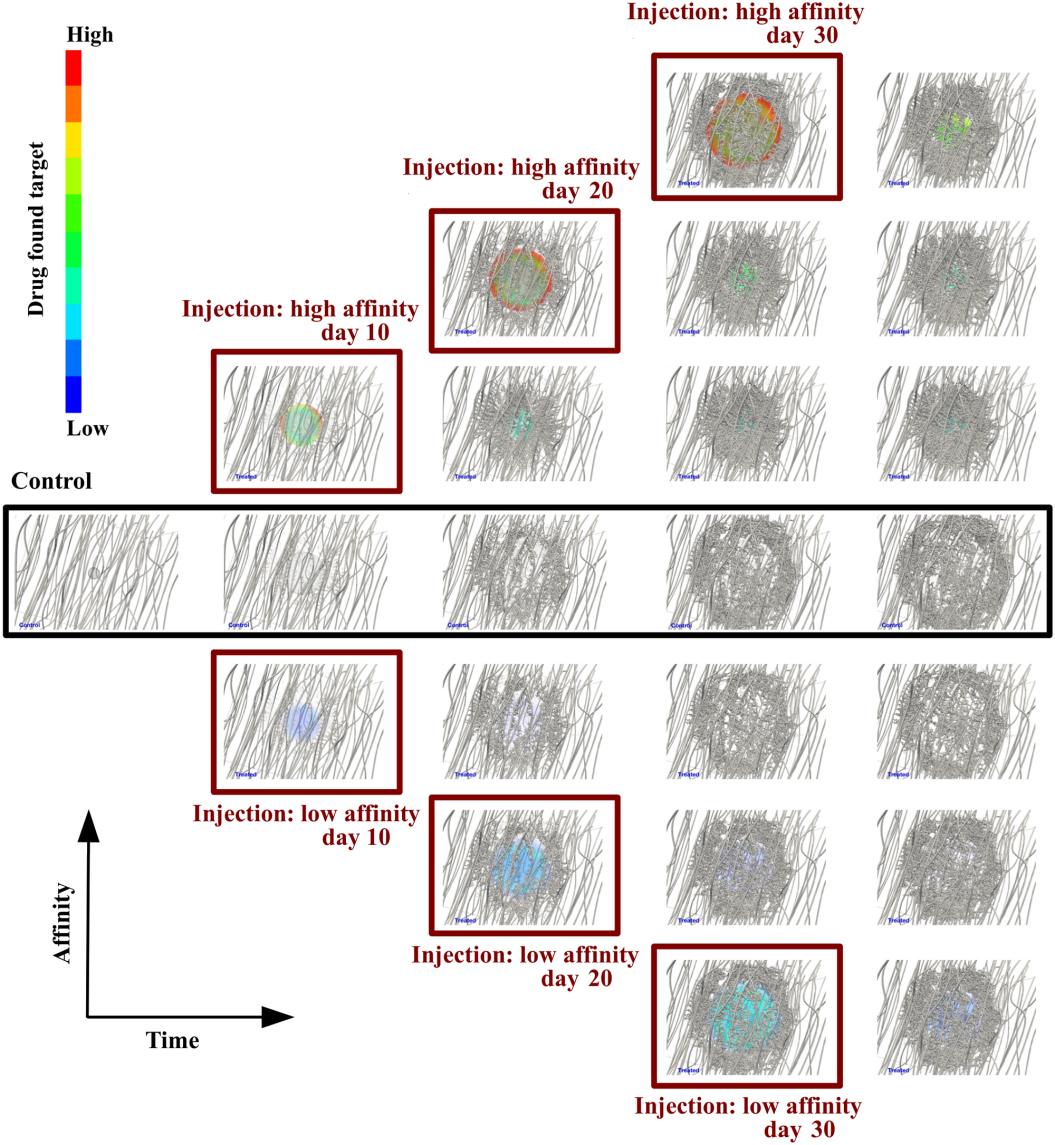
Snapshots of tumour growth and angiogenesis over time. Simulation results visualisation comparison of the control (centre row) versus the treated case for low poresize, *r*_p_, and two extreme affinity ratio, *k*_on_, values (0.005 s^-1^ and 5 s^-1^). From left to right, the snapshots at the second column correspond to day 11, the third column to day 21, the fourth column to day 31, and the last column to day 40. Note for low affinity (bottom three rows) the low drug concentration in the tumour, while for high affinity (top three rows), as expected, the significant concentration of the cytotoxic drug. Notably, drug distribution is very heterogeneous for early-stage injections due to the non-hierarchical structure of the immature tumour vessels, thus, supporting the argument of the the spatio-temporal variability of the vascular tree in mammary tumours. Also, comparing the top right snapshot with the bottom counterpart, the tumour is fairly more regressed for high drug affinity—see also Fig 3C and 3A respectively.

In support to the above observations, Fig 4 illustrates the in-silico predictions of the proposed framework, where the control is compared against the treated (for low poresize only) with respect to the two extreme values of the drug affinity ratio. It is striking to note in this figure that *(a)* the concentration of the drug that has “hit” the cancer mass is approximately fifteen times higher for when the affinity is increased by four orders of magnitude (see also S3 Fig). *(b)* Also, it is interesting to observe the direct effect of the tumour vascular tree non-hierarchical structure and pattern to the distribution of the drug in the cancer mass. For high affinity drug and early-stage drug administration, the concentration of the cytotoxic is enhanced at sparse locations of tumours—especially those where perfused newly-formed vessels are located adjacent to the tumour. On the contrary, for late-stage injection (e.g., day 30), the concentration of the cytotoxic is rather evenly distributed at the tumour periphery.

### Vessel poresize affects solid stress and IFP normalisation for low affinity but not high affinity cytotoxic drugs: Implications for staged delivery

**Fig 5:**
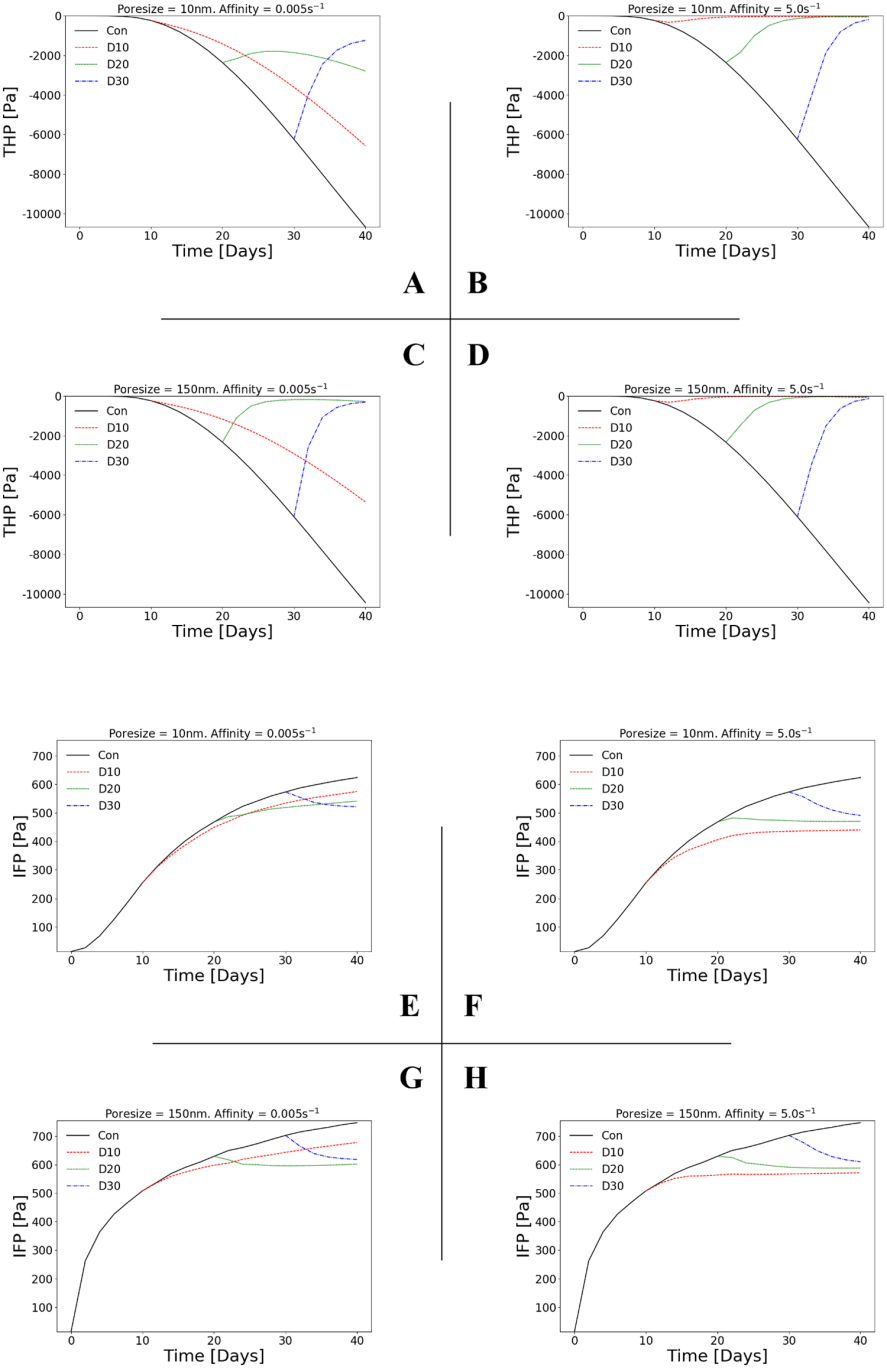
THP and IFP normalisation depends on vessel poresize for low affinity but not high affinity drugs. Line plots of (A—D) tissue hydrostatic pressure (THP), and (E—H) interstitial fluid pressure (IFP), as a function of time. Each 2×2 matrix of plots shows the results two poresizes and two affinities: *r*_p_ = 10 nm or 150 nm, and *k*_on_ = 0.005 s^-1^ or 5 s^-1^ respectively. Each line plot shows the in-silico predictions for the control and the treated, with drug injected at day 10 (D10), day 20 (D20) or day 30 (D30). See also S5 and S6 Figs for simulation results involving intermediate values of poresize and affinity. Negative THP in (A—D) denotes compressive stress.

The effects of binding affinity and vessel poresize on the tumour’s physical environment were investigated by plotting the tissue hydrostatic pressure (THP) of the solid stresses and the interstitial fluid pressure (IFP)—both evaluated at the tumour and the peri-tumoural host tissue—as a function of time for the four combinations of low and high affinity and poresize (Fig 5). As before, each plot depicts the control case and the treated with injections at day 10, day 20 or day 30 from baseline.

Both THP and IFP show similar trends as the tumour volume (Fig 5), reflecting their interdependency. The THP increases approximately linearly in the control case, reflecting the increase in solid stress due to tumour growth (Fig 5A). After injection, THP decreases with increasing affinity and poresize, with high affinity causing an almost complete alleviation of solid stress (i.e. THP = 0) independently of poresize or injection time (Fig 5B and 5D). Low affinity produces a more complex picture, which again mirrors the tumour volume: only late injection (day 30) can reduce the THP monotonically, with the earlier injections showing either monotonically increasing THP (day 10), or decreasing THP followed by relapse (Fig 5A and 5C).

In the control case, the IFP increases logarithmically for high poresize, which produces a sharper initial gradient and maximum value than for low poresize due to the increased extravasation flux. After injection, the maximum value of the IFP decreases with increasing affinity, and at high affinity converges to approximately the same value, independently of poresize or injection time (Fig 5F and 5H). At low affinity a similar trend to THP is predicted: only late injection (day 30) can reduce the IFP monotonically, with the earlier injections showing either monotonically increasing IFP (day 10), or decreasing IFP followed by relapse (Fig 5E and 5G).

Taken together, these results propose two main points: *(a)* THP and IFP are implicitly reduced by cytotoxic drug delivery, and *(b)* as a result of the reduced IFP, drugs that are dependent on convective transport—such as liposomes or nanoparticles—should not be administered after treatment by cytotoxic drugs. This has implications for therapies that aim to alleviate solid stresses in order to decompress collapsed vasculature and, hence, enhance drug delivery and for staged treatments that aim to maximise delivery of nanoparticles [13, 43].

### Vessel poresize affects vascular architecture normalisation for low affinity but not high affinity cytotoxic drugs

**Fig 6:**
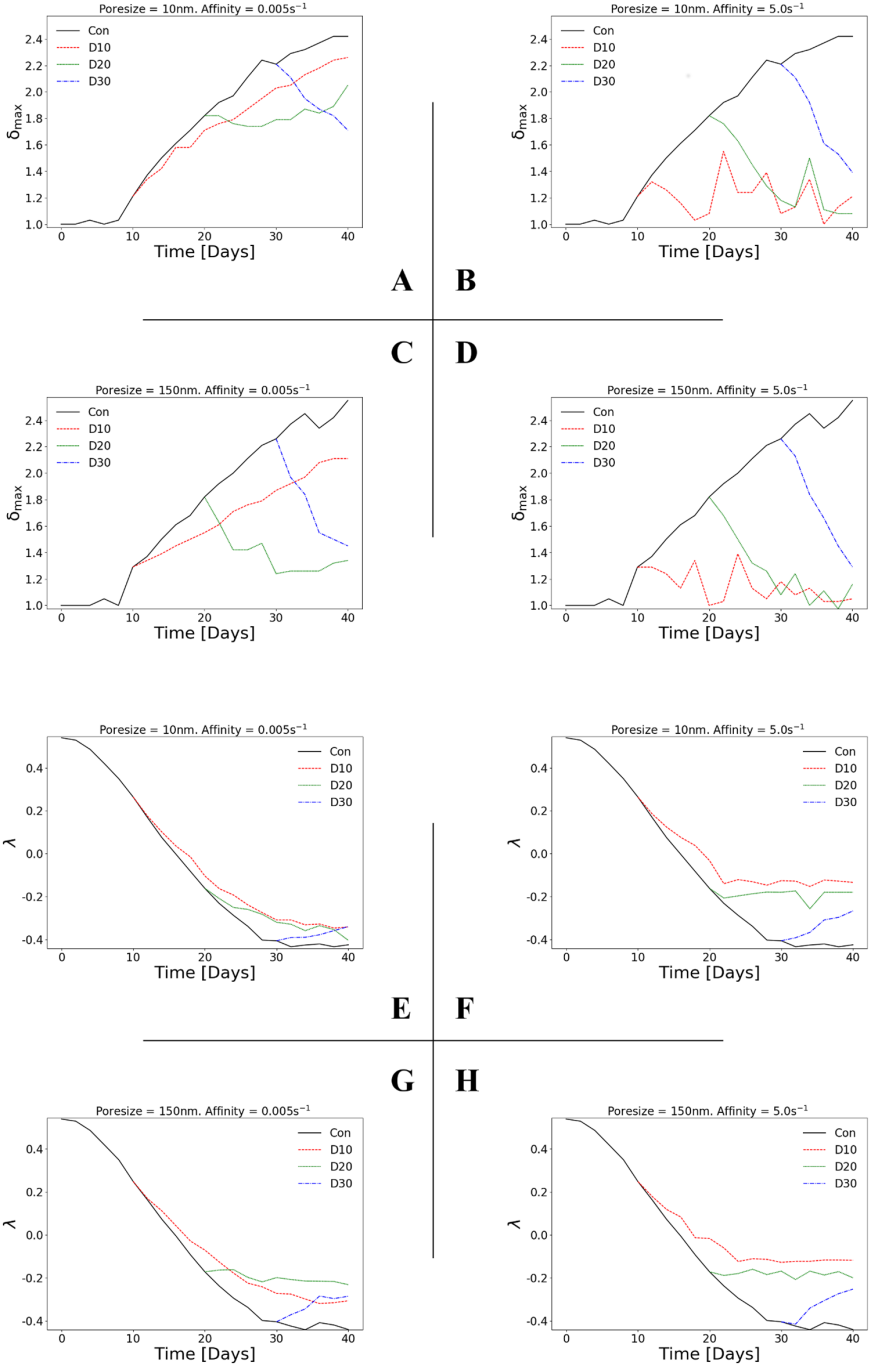
Vascular architecture normalisation depends on vessel poresize for low affinity but not high affinity drugs. Line plots of (A—D) maximum distance between adjacent vessels (*δ*_max_; normalised), and (E—H) vessel distribution convexity (λ), as a function of time. Each 2×2 matrix of plots shows the in-silico predictions for two poresizes and two affinities: *r*_p_ = 10 nm or 150 nm, and *k*_on_ = 0.005 s^-1^ or 5 s^-1^ respectively, while each plot depicts the results for the control and the treated, with drug injected at day 10 (D10), day 20 (D20) or day 30 (D30). See also S7 and S8 Figs for simulation results involving intermediate values of poresize and affinity.

To investigate the effect of cytotoxic drug delivery on tumour vessel architecture, Fig 6A–6D shows *δ*_max_ while Fig 6E–6H λ as a function of time for the four combinations of low and high affinity and poresize. As before, each plot shows the control case and injections at day 10, day 20 or day 30 from baseline.

In the control case, *δ*_max_ increases approximately linearly and in phase with the relative tumour volume increase, as can be seen by comparison of Figs 3A–3D and 6A–6D, respectively. This relationship with the tumour volume is also predicted after injection in all cases: *δ*_max_ increases with tumour volume until treatment, when it either continues to increase but to a smaller maximum (injection at day 10), or decreases after treatment (injections at day 20 or day 30). This is due to the development of compressive solid stress that results in larger avascular regions within the tumour. As a result, *δ*_max_ has a similar dependency on affinity and poresize: high affinity produces a more normalised vascular structure than low affinity, for all injection times (comparing columns in Fig 6A), and poresize only influences earlier injections (day 10 and day 20) at low affinity (comparing rows in Fig 6A).

The value of λ decreases smoothly from a positive to a negative value in the control case, which reflects the pathological change in distribution of the vasculature from a regular/uniform to an irregular/non-uniform pattern (e.g. Fig 6B). After injection λ becomes less negative (i.e. more physiological), with high affinity producing a more normal vasculature than low affinity (comparing columns in Fig 6B) and poresize only influencing the result at low affinity for an intermediate injection time at day 20 (comparing rows in Fig 6B). Considered together, the results for *δ*_max_ and λ indicate that *(a)* cytotoxic drugs can implicitly normalise the tumour-associated vasculature, and *(b)* this normalisation is only dependent on the size of the pores of the tumour vessels for low affinity drugs.

### Increasing cytotoxic drug affinity widens the window for vascular architecture normalisation: Methods to enhance delivery

**Fig 7:**
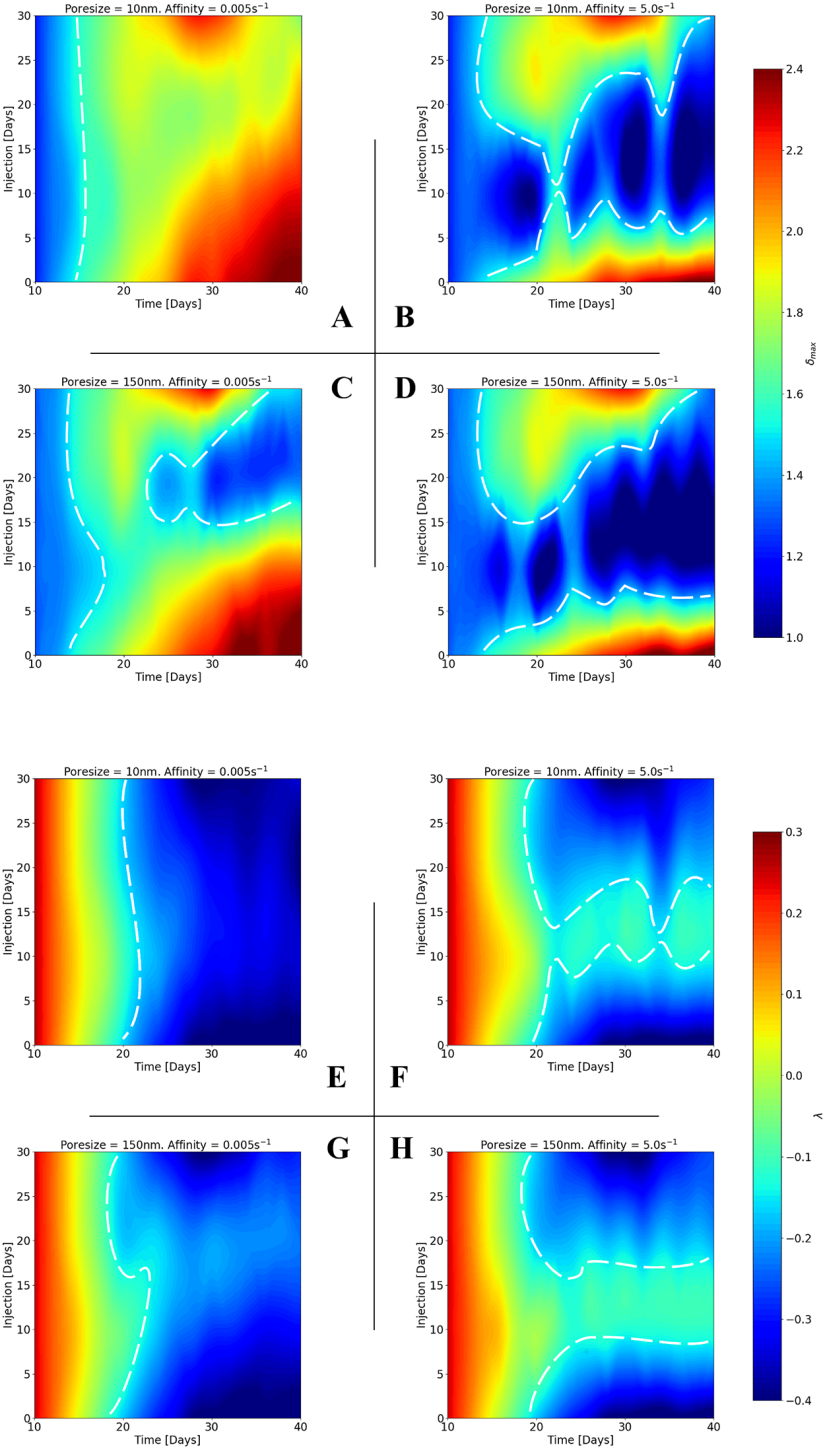
Increasing drug affinity widens the window for vascular normalisation. Contour plots of (A—D) maximum distance between adjacent vessels, *δ*_max_, and (E—H) vessel distribution convexity, λ, with respect to tumour development time and injection time. Each 2×2 matrix of contours depicts the results for two poresizes and two affinities: *r*_p_ = 10 nm or 150 nm, and *k*_on_ = 0.005 s^-1^ or 5 s^-1^ respectively. See also corresponding S9 and S10 Figs for simulation results involving intermediate values of poresize and affinity.

The optimal time period (‘window’) for vascular architecture normalisation is explored in Fig 7, which shows contour plots of (plates A—D) *δ*_max_, and (plates E—H) λ as a function of injection time and time from baseline for the four combinations of low and high affinity and poresize. The contours were calculated by cubic interpolation of the injection data (D10, D20, D30) presented in Fig 6 using SciPy’s interpolation module (*scipy.interpolate*).

As in the previous section, Fig 7A, 7C, 7E and 7G indicate that at low affinity, hyperpermeable tumour vessels have a potential normalising the vascular network structure (i.e. reduce *δ*_max_ to 1 and increase λ to positive values). This is evident in Fig 7C with the formation of the blue-coloured valley (bounded by the dashed white line) which designates that for high poresize and low drug affinity extravascular space becomes more organised, *δ*_max_ → 1; whereas in the corresponding Fig 7G for λ, the in-silico results do not suggest an improved vascular hierarchy—the parameter colour map is relatively insensitive to the time of injection.

On the contrary, by comparison of columns 1 and 2 in Fig 7, increasing the affinity of the chemotherapeutic agent both the extend of the vascular structure normalisation and the ‘window’ is significantly improved. The contours illustrate this effect graphically, i.e. cytotoxic drugs can normalise *δ*_max_ across time post the time of injection, while λ can be normalised only at a broad range of injection—especially in early and intermediate times (with respect to the tumour volume) of injection. Interestingly, as indicated by the regions bounded by the dashed lines, in Fig 7D a breadth of a dark blue region—which indicates *δ*_max_ approaching the physiological range, 1—1.4—orients the optimal window where the tumour vessels become relatively even spaced; while in Fig 7H the light green band—with λ converging towards physiological values, ≈ 0—indicates a strong tendency for the vascular re-organisation. This suggests that cytotoxic drugs can be used to implicitly normalise tumour-associated vasculature and are largely independent of the tumour’s stage.

### Combined high affinity drug and large vascular permeability can enhance cytotoxic drug delivery

**Fig 8:**
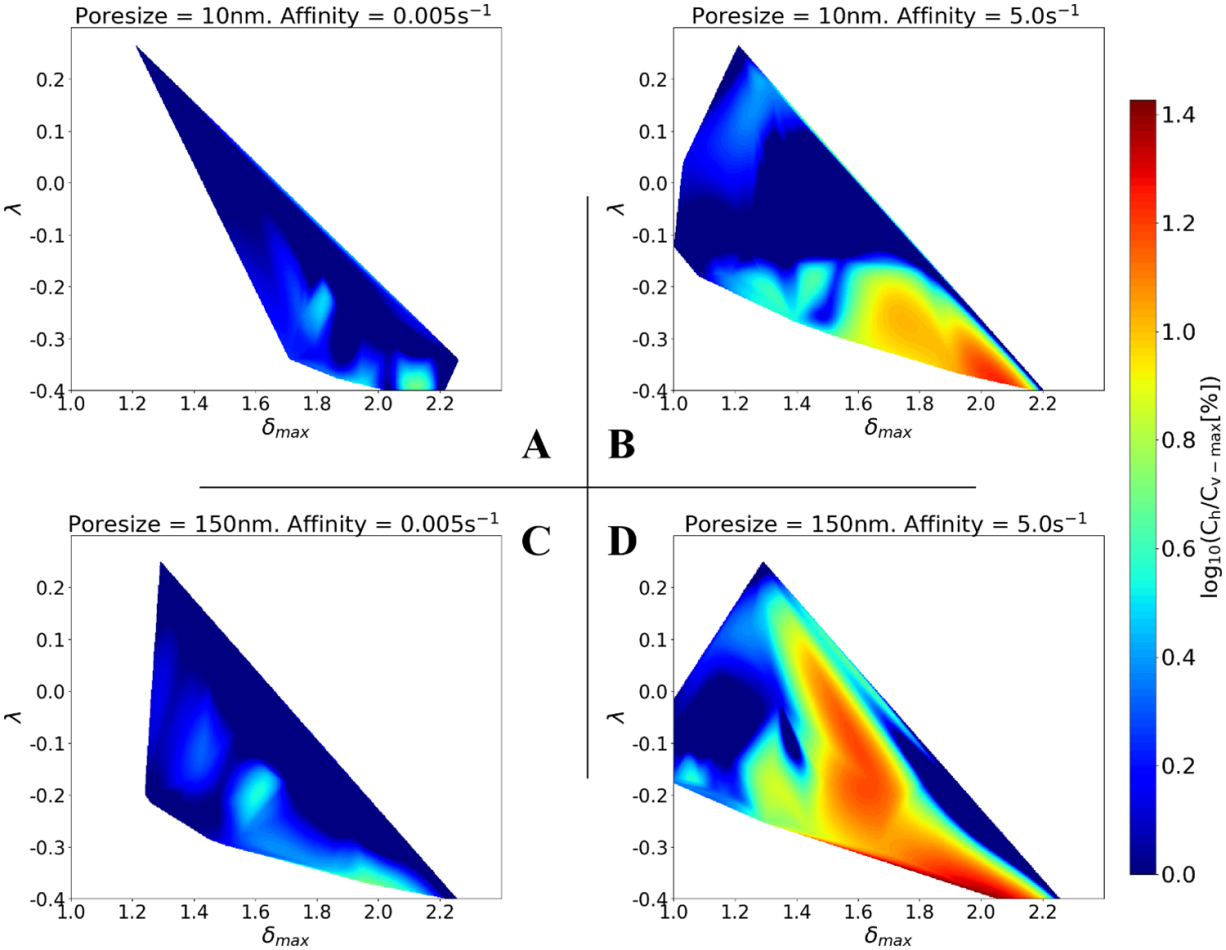
High drug affinity ratio and large vessel poresize increases delivery efficacy potential. Contour plots of the percentage of drug concentrated in the tumour, *c*_h_, as a function of vascular network structural parameters λ and *δ*_max_. Note that the common logarithm of *c*_h_ is taken, as *c*_h_ can vary by over a factor of 10 between the lowest and highest affinity ratios and poresize. The contours contained in the 2×2 matrix illustrates the in-silico results for two poresizes (A,B: *r*_p_ = 10 nm; C,D: *r*_p_ = 150 nm) and two affinities (A,C: *k*_on_ = 0.005 s^-1^; B,D: *k*_on_ = 5 s^-1^). See also S11 Fig for simulation results involving intermediate values of poresize and affinity.

To explicitly link vascular architecture with cytotoxic delivery efficiency and follow-up drug treatment potential, contour plots of *δ*_max_ versus λ with respect to the variable *c*_h_, which denotes the total (bound and internalised) cancer drug concentration, for the four combinations of low and high affinity and poresize are illustrated in Fig 8. The contours were calculated via cubic interpolation of the three variables using the above-mentioned interpolation tools of the SciPy library.

The range of *δ*_max_ and λ, i.e. the area of the contour, depends on poresize only at low affinity (compare rows in Fig 8). Also, comparing the columns in Fig 8, increased affinity expands the area of the contours irrespective of poresize. This supports the argument that a high-affinity drug has more potential normalising the architecture of the tumour vascular tree, which is reflected by the previous findings. Interestingly, the largest values of *c*_h_ are obtained for the most pathological vascular structures, i.e. large *δ*_max_ and negative λ as shown with dark red primarily in Fig 8D. Furthermore, the ‘window’ of treatment—referred here as the ‘hotspot’ region in the contours—can be increased substantially by increasing both affinity and poresize (see Fig 8A and 8D). However, by comparison of Fig 8B and 8D, we note that less permeable tumour vessels decrease the potential of the cytotoxic drugs to target the tumour—especially for when the vascular tree is less hierarchical (i.e. for negative convexity parameters) and less structured (i.e. increased vascular space; *δ*_max_ > 1.4). In summary, these results, and in conjunction with the extensive series of simulation results shown in S11 Fig, suggest that the combination of high poresize and high affinity allows the drug to target the tumour across a wide range of vessel architectures. Hence, we propose that blood vessel normalisation using chemical agents that could reinforce the endothelial wall barrier and reduce the size of intracellular fenestrations should be avoided prior to injecting cytotoxic drugs, as demonstrated in Fig 8A.

### Conclusion

We have presented a novel in-silico multiscale modelling framework of coupled tumour growth, angiogenesis and drug delivery. The model builds on our previous work [21], allowing for realistic simulations of in-vivo conditions that include: *(i)* dynamic remodelling of the tumour-associated vascular network as a result of growth-induced solid stresses, *(ii)* biophysical vessel sprouting that explicitly accounts for chemo-, hapto- and mechanotaxis, and *(iii)* solid stress-dependent vascular remodelling and compression/collapse. Here we extended the model to include extra- and intravascular drug delivery, and specified the model to simulate the transport of cytotoxic drugs to murine mammary carcinomas. The complete model has been implemented in our in-house, open-source numerical platform FEB3 (https://bitbucket.org/vasvav/feb3-finite-element-bioengineering-in-3d/wiki/Home). The proposed in-silico framework allowed us to study the dynamics of tumour growth and cytotoxic drug delivery, and hence comment on the following key topics identified by [39]: *(a)* the relative roles of convective and diffusive transport for cytotoxic drugs, *(b)* the effects of tumour microvascular structure and function, and *(c)* methods to enhance delivery by modification of IFP and reduction of tissue stress by induction of tumour cell apoptosis.

Furthermore, our model is flexible: drugs of varying size and affinity can be simulated, while the poresize of the tumour vessels can be specified. We can thus implicitly simulate the effect of neoadjuvant blood vessel normalisation by setting a small initial vessel wall poresize. This allowed us to also comment on, firstly, the relative importance of affinity and poresize in targeting tumours, and subsequent normalisation of vasculature structure. Secondly, the in-silico model permitted us to investigate the potential implications for staged delivery (viz. normalisation of THP and IFP).

The main hypotheses proposed by our model are: *(i)* confirm that chemotherapeutic agents’ delivery is dominated by diffusive transport; *(ii)* the time of treatment is important for low affinity but not high affinity drugs; *(iii)* vessel poresize plays an important role in the effect of low affinity but not high affinity cytotoxic drugs; *(iv)* high affinity cytotoxic drugs provide a large window for vascular architecture normalisation; and *(v)* the combination of large poresize and high affinity enhances cytotoxic drug delivery efficiency. The model provides valuable insight into the complex system of biophysical factors that generate these hypotheses. In particular, it suggests that the combination of diffusive transport and pathological tumour-associated angiogenesis allows cytotoxic drugs to target the tumour across a broad range of vasculature architectures. Furthermore, our modelling framework allows for these hypotheses to be tested by comparing directly to experimental data from mouse models treated with vascular normalisation agents and drugs of varying affinity, when it becomes available.

We note that it is difficult to directly validate our drug delivery model predictions, given the sparsity of literature available that is both suitable in terms of the application and that can completely specify the model. Here we have made every effort to specify the model where the data were available; this is evidenced in our previous work [21], where we tested every component of the model, except the drug delivery module, against in vivo experimental data of murine tumours. We can, however, qualitatively compare our predictions to similar experimental work, such as [38, 44], who observed that structural vascular normalisation lowered interstitial fluid pressure in murine models and, hence, subsequent nanoparticle penetration from the vessels into the tumour tissue has been improved. This is in agreement with our prediction of smaller vascular pore sizes producing lower interstitial fluid pressure. Furthermore, it was found in Chauhan et al. [38] that chemotherapy delivery is optimised at pore sizes in the order of 150 nm and drops for smaller pore sizes, which also agrees with our model predictions that recommend that 150 nm, not smaller, pore sizes are more suitable for effective chemotherapeutic drug delivery.

While we have strived to make the model representative of in-vivo conditions, it has limitations. The high complexity of the model requires a large number of model parameters to be specified and, hence, the results are dependent upon the choice of these parameters. Where possible we chose experimental data from the literature to best represent the growth of murine mammary carcinomas, detailed in S4 Table. Furthermore, as previously mentioned, we have already validated the coupled tumour angiogenesis and growth model against experimental data in previous work [21]. As such, while the choice of the drug delivery model parameters affect the result quantitatively, we expect the qualitative in-silico predictions to remain the same.

In order to reduce the dependency of the model on material parameters, and in particular those where the literature is sparse, some simplifications were made. The tissue hydraulic conductivity was assumed isotropic and independent of solid deformations, which would affect fluid flow as the tumour grows [34, 45]. Here however we have simulated a spherical tumour into a homogeneous matrix and, thus, can reasonably expect that this assumption would only change primarily the magnitude and not the direction of IFV. We did not model the deposition of new collagen by tumour cells, which in turn would affect the matrix composition and interstitial hydraulic conductivity. Again, due to the symmetric nature of the problem, we expect this to only affect the magnitude of the results. The dynamic viscosity of blood and interstitial fluid is assumed constant and rate-independent, which, given the heterogeneous nature of the developing vascular network, could be expected to affect both the magnitude and direction of the fluid flow. However, as we have focused on cytotoxic drugs—which are more dependent on diffusive than convective transport—it is reasonable to ignore this effect. Finally, the model does not account for lymph-angiogenesis and the lymphatic vessels are not modelled explicitly, i.e. no description of lymphatic biomechanics, such as compression and collapse. This would be more important for larger drugs than those studied here, where the convective component needs to be modelled realistically to account for drainage and retention effects [46]. The study of larger drugs, such as liposomes, micella or drug-borne nanoparticles, is the subject of future work.

## Supporting information

S1 File In-silico cancer modelling framework implementation. (PDF)

S2 File Cytotoxic drug and cancer regression model sensitivity analysis. (PDF)

S1 Fig Three-dimensional finite element mesh of the tumour–host tissue domain and the one-dimensional finite element mesh of the vascular network. (A) Clipped mesh, showing the internal structure of the grid. (B) The extracted tumour region, shown here as a spheroid, with the complete vascular tree rendered as red segments and the black points denoting the vascular nodes.
(TIF)

S2 Fig Time plots of the interstitial fluid velocity (IFV) magnitude. Line plots of the averaged IFV magnitude as a function of time. The 3×4 matrix of plots depicts the in-silico results (both the control and the treated cases) for three poresizes and four affinities: *r*_p_ = 10 nm, 50 nm or 150 nm, and *k*_on_ = 0.005 s^-1^, 0.05 s^-1^, 0.5 s^-1^ or 5 s^-1^, respectively. All sub-figures illustrate the predictions for the control and the treated case (drug injected at day 10 (D10), day 20 (D20) or day 30 (D30)).
(TIF)

S3 Fig Time plots of the bound/associated drug and the internalised drug concentration. Line plots of the (A—D) bound/associated drug concentration, *c*_b_, and (E—H) internalised drug concentration, *c*_i_, expressed in dimensionless form (with respect to the injected drug concentration, *c*_v-max_) as a function of time. Each 2×2 matrix of plots depicts the in-silico results (treated case) for two poresizes: *r*_p_ = 10 nm or 150 nm, and two affinities: *k*_on_ = 0.005 s^-1^ or 5 s^-1^.
(TIF)

S4 Fig Time plots of the relative tumour volume development. Line plots of the relative tumour volume (*V* = Vol.(*t*)/Vol.(*t* = 0)−1) as a function of time. The 3×4 matrix of plots depicts the in-silico results (both the control and the treated cases) for three poresizes and four affinities: *r*_p_ = 10 nm, 50 nm or 150 nm, and *k*_on_ = 0.005 s^-1^, 0.05 s^-1^, 0.5 s^-1^ or 5 s^-1^, respectively. All sub-figures illustrate the predictions for the control and the treated case (drug injected at day 10 (D10), day 20 (D20) or day 30 (D30)).
(TIF)

S5 Fig Time plots of the tissue hydrostatic pressure (THP). Line plots of THP as a function of time. The 3×4 matrix of plots depicts the in-silico results (both the control and the treated cases) for three poresizes and four affinities: *r*_p_ = 10 nm, 50 nm or 150 nm, and *k*_on_ = 0.005 s^-1^, 0.05 s^-1^, 0.5 s^-1^ or 5 s^-1^, respectively. All sub-figures illustrate the predictions for the control and the treated case (drug injected at day 10 (D10), day 20 (D20) or day 30 (D30)).
(TIF)

S6 Fig Time plots of the interstitial fluid pressure (IFP). Line plots of IFP as a function of time. The 3×4 matrix of plots depicts the in-silico results (both the control and the treated cases) for three poresizes and four affinities: *r*_p_ = 10 nm, 50 nm or 150 nm, and *k*_on_ = 0.005 s^-1^, 0.05 s^-1^, 0.5 s^-1^ or 5 s^-1^, respectively. All sub-figures illustrate the predictions for the control and the treated case (drug injected at day 10 (D10), day 20 (D20) or day 30 (D30)).
(TIF)

S7 Fig Time plots of the maximum distance between adjacent vessels. Line plots of normalised *δ*_max_ as a function of time. The 3×4 matrix of plots depicts the in-silico results (both the control and the treated cases) for three poresizes and four affinities: *r*_p_ = 10 nm, 50 nm or 150 nm, and *k*_on_ = 0.005 s^-1^, 0.05 s^-1^, 0.5 s^-1^ or 5 s^-1^, respectively. All sub-figures illustrate the predictions for the control and the treated case (drug injected at day 10 (D10), day 20 (D20) or day 30 (D30)).
(TIF)

S8 Fig Time plots of the vascular network convexity. Line plots of λ as a function of time. The 3×4 matrix of plots depicts the in-silico results (both the control and the treated cases) for three poresizes and four affinities: *r*_p_ = 10 nm, 50 nm or 150 nm, and *k*_on_ = 0.005 s^-1^, 0.05 s^-1^, 0.5 s^-1^ or 5 s^-1^, respectively. All sub-figures illustrate the predictions for the control and the treated case (drug injected at day 10 (D10), day 20 (D20) or day 30 (D30)).
(TIF)

S9 Fig Heat maps of the vascular network maximum distance parameter with respect to tumour development time versus injection time. Contour plots of *δ*_max_ as a function of time and injection time. The 3×4 matrix of plots depicts the in-silico results for three poresizes and four affinities: *r*_p_ = 10 nm, 50 nm or 150 nm, and *k*_on_ = 0.005 s^-1^, 0.05 s^-1^, 0.5 s^-1^ or 5 s^-1^, respectively.
(TIF)

S10 Fig Heat maps of the vascular network convexity parameter with respect to tumour development time versus injection time. Contour plots of λ as a function of time and injection time. The 3×4 matrix of plots depicts the in-silico results for three poresizes and four affinities: *r*_p_ = 10 nm, 50 nm or 150 nm, and *k*_on_ = 0.005 s^-1^, 0.05 s^-1^, 0.5 s^-1^ or 5 s^-1^, respectively.
(TIF)

S11 Fig Heat maps of the cancer drug concentration with respect to tumour development time versus injection time. Contour plots of *c*_h_ as a function of tumour development time and injection time. The 3×4 matrix of plots depicts the in-silico results for three poresizes and four affinities: *r*_p_ = 10 nm, 50 nm or 150 nm, and *k*_on_ = 0.005 s^-1^, 0.05 s^-1^, 0.5 s^-1^ or 5 s^-1^, respectively.
(TIF)

S12 Fig Time plots of the fraction of perfused (tumour) vessels. Line plots of the fraction of perfused vessels (FPV) as a function of time. FPV is described as the ratio of the length of all functional vessels that are sufficiently perfused (blood flow velocity is >0.1 mm s^-1^) to the length of all functional vessels (i.e. that have not collapsed). The 3×4 matrix of plots depicts the in-silico results (both the control and the treated cases) for three poresizes and four affinities: *r*_p_ = 10 nm, 50 nm or 150 nm, and *k*_on_ = 0.005 s^-1^, 0.05 s^-1^, 0.5 s^-1^ or 5 s^-1^, respectively. All sub-figures illustrate the predictions for the control and the treated case (drug injected at day 10 (D10), day 20 (D20) or day 30 (D30)).
(TIF)

S1 Video In-silico tumour development simulation of the control and treated case for low-affinity drug injection at day 10. Side-by-side comparison of a control and a treated murine mammary carcinoma after a bolus injection (at day 10) of a cancer cytotoxic agent. The simulation features a tumour vasculature of *r*_*p*_ = 10 nm poresize, molecule (drug) size about *s*_c_ = 0.5 nm, and drug affinity rate *k*_on_ = 0.005 s^-1^. The wireframe on the left-hand-side depicts the tumour boundary and the coloured cloud on the right-hand-side corresponds to the distribution of the (dimensionless) drug concentration inside the tumour. The vascular network is shown as grey tubes on both sides of the animation, while the 3D tissue domain is made transparent for illustration purposes.
(MP4)

S2 Video In-silico tumour development simulation of the control and treated case for low-affinity drug injection at day 20. Side-by-side comparison of a control and a treated murine mammary carcinoma after a bolus injection (at day 20) of a cancer cytotoxic agent. The simulation features a tumour vasculature of *r*_*p*_ = 10 nm poresize, molecule (drug) size about *s*_c_ = 0.5 nm, and drug affinity rate *k*_on_ = 0.005 s^-1^. Refer to the description of S1 Video. for interpretation of the animation features.
(MP4)

S3 Video In-silico tumour development simulation of the control and treated case for low-affinity drug injection at day 30. Side-by-side comparison of a control and a treated murine mammary carcinoma after a bolus injection (at day 30) of a cancer cytotoxic agent. The simulation features a tumour vasculature of *r*_*p*_ = 10 nm poresize, molecule (drug) size about *s*_c_ = 0.5 nm, and drug affinity rate *k*_on_ = 0.005 s^-1^. Refer to the description of S1 Video. for interpretation of the animation features.
(MP4)

S4 Video In-silico tumour development simulation of the control and treated case for high-affinity drug injection at day 10. Side-by-side comparison of a control and a treated murine mammary carcinoma after a bolus injection (at day 10) of a cancer cytotoxic agent. The simulation features a tumour vasculature of *r*_*p*_ = 10 nm poresize, molecule (drug) size about *s*_c_ = 0.5 nm, and drug affinity rate *k*_on_ = 5 s^-1^. Refer to the description of S1 Video. for interpretation of the animation features.
(MP4)

S5 Video In-silico tumour development simulation of the control and treated case for high-affinity drug injection at day 20. Side-by-side comparison of a control and a treated murine mammary carcinoma after a bolus injection (at day 20) of a cancer cytotoxic agent. The simulation features a tumour vasculature of *r*_*p*_ = 10 nm poresize, molecule (drug) size about *s*_c_ = 0.5 nm, and drug affinity rate *k*_on_ = 5 s^-1^. Refer to the description of S1 Video. for interpretation of the animation features.
(MP4)

S6 Video In-silico tumour development simulation of the control and treated case for high-affinity drug injection at day 30. Side-by-side comparison of a control and a treated murine mammary carcinoma after a bolus injection (at day 30) of a cancer cytotoxic agent. The simulation features a tumour vasculature of *r*_*p*_ = 10 nm poresize, molecule (drug) size about *s*_c_ = 0.5 nm, and drug affinity rate *k*_on_ = 5 s^-1^. Refer to the description of S1 Video. for interpretation of the animation features.
(MP4)

S1 Table Fluid mechanics model parameters. List of model parameters associated with the *Fluid Solver Module* (see Fig 1). Cells marked with an asterisk denote shared values for both tissue types, while “VSC” denotes blood vessel and “LMP” denotes the lymphatic vessel.
(PDF)

S2 Table Vascular network model parameters. List of model parameters associated with the *Vascular Network Module* (see Fig 1). Parameters with a star (⋆) correspond to non-perfused or hypo-perfused vessels, while those with a dagger (†) correspond to well-perfused vessels. The parameters with a double dagger (‡) denote the pre-set parameter values of the original vascular network, while the cell marked with an asterisk denotes shared value for both tissue types.
(PDF)

S3 Table Biochemical and drug delivery model parameters. List of model parameters associated with the *Biochemical Solver Module* and the *Drug Delivery Solver Module* (see Fig 1). Cells marked with an asterisk denote shared values for both tissue types, while “NA” denotes non-applicable.
(PDF)

S4 Table Solid mechanics model parameters. List of model parameters associated with the *Solid Solver Module* (see Fig 1). Cells marked with an asterisk denote shared values for both tissue types, while “NA” denotes non-applicable.
(PDF)

S5 Table Tumour volume development time rates. The tabulated data compare the tumour volume rates (in mm^3^ day^-1^) of the control versus the treated cases (for the three injection instants). Each sheet lists the results for a different pair of poresize and affinity values (A: *r*_p_ = 10 nm, *k*_on_ = 0.005 s^-1^; B: *r*_p_ = 10 nm, *k*_on_ = 5 s^-1^; C: *r*_p_ = 150 nm, *k*_on_ = 0.005 s^-1^; D: *r*_p_ = 150 nm, *k*_on_ = 5 s^-1^).
(XLSX)
