# Correction: In-silico dynamic analysis of cytotoxic drug administration to solid tumours: Effect of binding affinity and vessel permeability

**DOI:** 10.1371/journal.pcbi.1006880

**Published:** 2019-03-04

**Authors:** Vasileios Vavourakis, Triantafyllos Stylianopoulos, Peter A. Wijeratne

There is a funder missing from the Funding statement. The full statement should read: “VV has been partially supported by an FP7-PEOPLE project (Project ID: 627025; URL: https://cordis.europa.eu/project/rcn/188072_en.html), and TS has been supported by an FP7-IDEAS-ERC project (Project ID: 336839; URL: https://cordis.europa.eu/project/rcn/109567_en.html). VV has been also financially supported by an EPSRC-funded CMIC Platform Grant (Project ID: EP/M020533/1; URL: https://gow.epsrc.ukri.org/NGBOViewGrant.aspx?GrantRef=EP/M020533/1). This work was partly supported by H2020-WIDESPREAD-04-2017-Teaming Phase 1, Grant Agreement 763781, Integrated Precision Medicine Technologies. However, the funders had no role in the study design, data collection and analysis, the decision to publish, or the preparation of the manuscript. PAW has received no specific funding for this work.”
