# Supplementary material for: In-silico dynamic analysis of cytotoxic drug administration to solid tumours: Effect of binding affinity and vessel permeability
Source: PLoS Comput Biol. 2018 Oct 8;14(10):e1006460. doi: 10.1371/journal.pcbi.1006460 (PMC6193741; doi:10.1371/journal.pcbi.1006460)
Supplement: S1 File — (PDF) [file pcbi.1006460.s001.pdf]

SUPPORTING INFORMATION

In-silico Dynamic Analysis of Cytotoxic Drug Administration to Solid Tumours: Effect of Binding Affinity and Vessel Permeability

Vasileios Vavourakis, Triantafyllos Stylianopoulos, Peter A. Wijeratne

In-silico cancer modelling framework implementation

The in-silico cancer modelling framework has been implemented in a scalable C++ code, and incorporated into the existing in-house numerical analysis framework Finite Element Bioengineering in 3D (*FEB3*). *FEB3* is freely available upon request from: `https://bitbucket.org/vasvav/feb3-finite-element-bioengineering-in-3d/wiki/Home`. *FEB3* has the capacity to simulate coupled multiphysics, multiscale tissue (solid and fluid) biomechanics problems using the finite element method or/and the isogeometric analysis method. Most recent publications by the authors that utilise the in-silico platform include: [1–4]

FEB3 has been designed in an object-oriented manner and facilitates parallel computations using the message passing interface library *MPICH*, while it is founded on the following high-performance, open-source numerical libraries and tools:

- *blitz++* is a meta-template library in C++ which was utilised in *FEB3* for tensor algebra and multi-dimensional tensor manipulation [5].

- *GNU Scientific Library* (`https://www.gnu.org/software/gsl/`) is an ANSI-C library that contains an wide range of mathematical routines (over 1000 in total) such as random number generators, special functions, statistics, numerical differentiation, data fitting, etc. [6]. More details about the library can be found in the online documentation: `https://www.gnu.org/software/gsl/manual/html_node/`.

- *METIS* (`http://glaros.dtc.umn.edu/gkhome/metis/metis/overview`) and *ParMETIS* (`http://glaros.dtc.umn.edu/gkhome/metis/parmetis/overview`) is a pair of libraries containing established algorithms for partitioning graphs, partitioning finite element meshes and producing fill-reducing orderings for sparse matrices, in serial and in parallel computing respectively [7]. Both the three-dimensional finite element tissue mesh and the one-dimensional vascular network mesh was decomposed and parallelised across multiple processors using *ParMETIS* library.

- *MPICH* (`https://www.mpich.org/`) is a standardised and portable message-passing system which works as a communication protocol for parallel computing in multiprocessor computers / computer clusters.

- *PETSc* (`https://www.mcs.anl.gov/petsc/`) is a suite of data structures and routines for the solution of scientific applications, and is been used within *FEB3* in solving linear and nonlinear systems [8, 9]. *PETSc* is also integrated with *MPICH* to facilitate parallel computations, while it communicates with *METIS* and *ParMETIS* for sparse system partitioning.

- *libMesh* (`http://libmesh.github.io/`) is the top-level library used by *FEB3*. *libMesh* is an object-oriented C++ framework for the numerical simulation of partial differential equations using arbitrary unstructured discretisations on serial and parallel platforms [10], while it integrates with high-performance computing libraries such as *PETSc*.
- *Gmsh* (`http://gmsh.info/`) is an open-source platform for finite element mesh generation and finite element analysis post-processing that has a built-in computer-aided design engine [11]. *Gmsh* is being used by *FEB3* to supply three-dimensional meshes of the tissue/extracellular matrix domain.
- *Paraview* (`https://www.paraview.org/`) is an open-source, multi-platform data analysis and visualization application. *FEB3* exports all simulation results – compatible with the extensible markup language (XML) format – which are in turn compatible and supported with the data formatting used in *Paraview*.

All simulations presented in this work were carried out on a Linux (kernel version: *3.13.0-71-generic*) desktop machine having an Intel Xeon E5-2620 CPU (@2.0 GHz $\times$ 6) and 16 GB (@1333.33 MHz DDR3) RAM memory, and on a Mac Pro desktop machine having an Intel Xeon E5 (@3.0 GHz $\times$ 8) and 64 GB (@1866.67 MHz DDR3) RAM memory.

References

1. Wijeratne PA, Vavourakis V, Hipwell JH, Voutouri C, Papageorgis P, Stylianopoulos T, et al. Multiscale modelling of solid tumour growth: the effect of collagen micromechanics. Biomechanics and Modeling in Mechanobiology. 2015; p. 1–12. doi:10.1007/s10237-015-0745-2.
2. Vavourakis V, Eiben B, Hipwell JH, Williams NR, Keshtgar M, Hawkes DJ. Multiscale Mechano-Biological Finite Element Modelling of Oncoplastic Breast Surgery–Numerical Study towards Surgical Planning and Cosmetic Outcome Prediction. PLoS ONE. 2016;11(7):e0159766. doi:10.1371/journal.pone.0159766.
3. Vavourakis V, Wijeratne PA, Shipley R, Loizidou M, Stylianopoulos T, Hawkes DJ. A Validated Multiscale In-Silico Model for Mechano-sensitive Tumour Angiogenesis and Growth. PLOS Computational Biology. 2017;13(1):e1005259. doi:10.1371/journal.pcbi.1005259.
4. Wijeratne PA, Hipwell JH, Hawkes DJ, Stylianopoulos T, Vavourakis V. Multiscale biphasic modelling of peritumoural collagen microstructure: The effect of tumour growth on permeability and fluid flow. PLoS ONE. 2017;12(9):e0184511. doi:https://doi.org/10.1371/journal.pone.0184511.
5. Veldhuizen T. Blitz++ User's Guide: A C++ class library for scientific computing; 2006. Available from: `http://blitz.sourceforge.net/resources/blitz-0.9.pdf`.
6. Galassi M, Davies J, Thailer J, Gough B, Jungman G, Alken P, et al.. GNU Scientific Library Reference Manual; 2013. Available from: `http://www.gnu.org/software/gsl/`.
7. Karypis G, Kumar V. MeTis: Unstructured Graph Partitioning and Sparse Matrix Ordering System, Version 4.0; 2009. `http://www.cs.umn.edu/~metis`.

8. Balay S, Gropp WD, McInnes LC, Smith BF. Efficient Management of Parallelism in Object Oriented Numerical Software Libraries. In: Arge E, Bruaset AM, Langtangen HP, editors. Modern Software Tools in Scientific Computing. Birkhäuser Press; 1997. p. 163–202.

9. Balay S, Abhyankar S, Adams MF, Brown J, Brune P, Buschelman K, et al. PETSc Users Manual. Argonne National Laboratory; 2014. ANL-95/11 - Revision 3.5. Available from: `http://www.mcs.anl.gov/petsc`.

10. Kirk BS, Peterson JW, Stogner RH, Carey GF. `libMesh`: A C++ Library for Parallel Adaptive Mesh Refinement/Coarsening Simulations. Engineering with Computers. 2006;22(3–4):237–254.

11. Geuzaine C, Remacle J. Gmsh: A 3-D finite element mesh generator with built-in pre- and post-processing facilities. International Journal for Numerical Methods in Engineering. 2009;79(11):1309–1331. doi:10.1002/nme.2579.
