# Supplementary material for: In-silico dynamic analysis of cytotoxic drug administration to solid tumours: Effect of binding affinity and vessel permeability
Source: PLoS Comput Biol. 2018 Oct 8;14(10):e1006460. doi: 10.1371/journal.pcbi.1006460 (PMC6193741; doi:10.1371/journal.pcbi.1006460)
Supplement: S2 File — (PDF) [file pcbi.1006460.s002.pdf]

SUPPORTING INFORMATION

In-silico Dynamic Analysis of Cytotoxic Drug Administration to Solid Tumours: Effect of Binding Affinity and Vessel Permeability

Vasileios Vavourakis, Triantafyllos Stylianopoulos, Peter A. Wijeratne

Cytotoxic drug and cancer regression model sensitivity analysis

Biomechanically-associated tumour development model parameters

The effect of the exponent δ_g on the growth of the solid tumour through function $G(\epsilon) = \alpha_g \, \epsilon^{\delta_g}$ (see also Eq (14) and parameters in S4 Table) is tested here. The baseline simulations presented in the main body of the paper use a cubic polynomial for the growth function, i.e. $\delta_g = 3$. Thus, we run simulations (both control and treated cases) where G is a linear function of the state variable ϵ, which represents the extracellular matrix (ECM) composition and "structural integrity;" in this case $\delta_g = 1$. Also, we run the same set of simulations with $\delta_g = 9$. Intuitively, in the latter case, the magnitude of the cancer mass growth, G, is very sensitive to reductions of the extracellular matrix, ϵ. All test simulations were run for tumour vessels' poresize $r_p = 50$ nm, and drug binding rate (affinity) $k_{on} = 0.05$ s^{-1}.

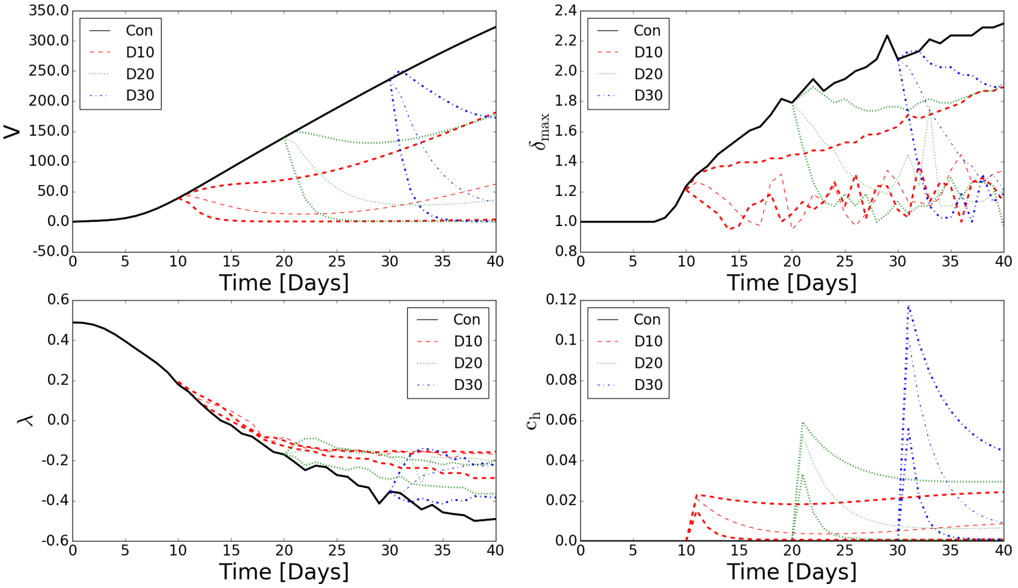

Fig 1. Impact of the δ_g parameter on tumour volume, vascular network structure and drug concentration predictions.

Line plots (from top to bottom and from left to right) of the relative tumour volume ($V = {}^{\text{Vol.}(t)}/_{\text{Vol.}(t=0)} - 1$), the vascular network structural parameters δ_{max} and λ, and the drug concentrated in the tumour, c_h, as a function of time. Plots with the baseline values are shown with a thin line. Poresize and drug affinity were fixed at $r_p = 50$ nm and $k_{on} = 0.05$ s^{-1} respectively.

Figure 1 shows time plots of the relative tumour volume development (both for the control and the treated cases at three injection times), the vascular network structural parameters $\delta_{\max}$ and λ (as described in detail by Baish et al. [1]), and the drug concentration in the tumour region, c_{h}. As seen in the volume time-plots, for a linear function of G, tumour relapse and growth is much faster in all treated cases when compared to the baseline treated simulations (thin dashed lines). Whereas, as expected in the 9th-order case, the tumour regresses rapidly due to the degradation of the tumour ECM in effect of the cancer drug (see Eq (12) and Eq (13)). Also, the time-plots of the maximum distance of adjacent blood vessels of the micro-vascular tree, $\delta_{\max}$, for when $\delta_{\mathrm{g}} = 3$ (baseline) are comparable to the treated simulations when $\delta_{\mathrm{g}} = 9$, while for $\delta_{\mathrm{g}} = 1$ the tumour continues growing (at slower rate) hence repelling and compressing surrounding tumour vessels.

Subsequently, we tested the effect of the constant parameter a_{w}, which is used in the biomechanical stiffness function: $m(\epsilon) = \mu\, \epsilon^{a_{\mathrm{w}}}$, which is in turn introduced in the stored-energy potential function $\bar{W}$ (see Eq (15)). In the baseline simulations reported in the main body of the manuscript, function m has linear dependence on the extracellular matrix state variable ϵ (i.e. $a_{\mathrm{w}} = 1$). However, to examine the sensitivity of the biomechanical variable m in the tumour development predictions with respect to the dynamics of ϵ (see Eq (12)), as a consequence of the cancer stroma depletion from the drug and the peritumoural stroma from the presence of the matrix degrading enzymes (secreted from the tip ECs and tumour cells; see Eq (14) from [2]), the parameter a_{w} was allowed to take values 0.5 and 2.0.

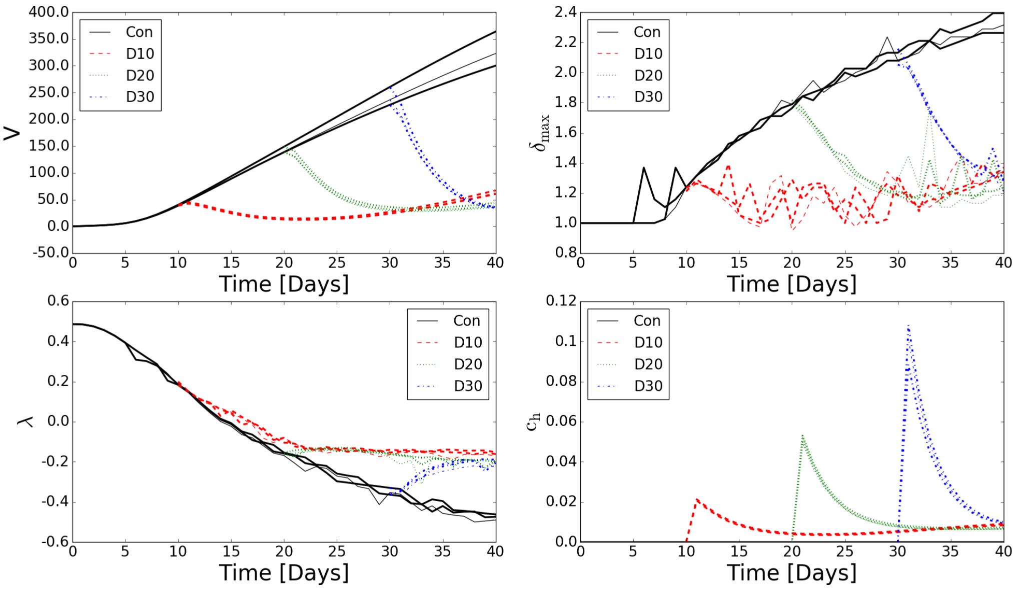

Fig 2. Impact of the a_{w} parameter on the tumour volume, vascular network structure and drug concentration predictions.

Line plots (from top to bottom and from left to right) of the relative tumour volume, V, the vascular network structural parameters $\delta_{\max}$ and λ, and the drug concentrated in the tumour, c_{h}, as a function of time. Plots with the baseline values are shown with a thin line. Poresize and drug affinity were fixed at $r_{\mathrm{p}} = 50$ nm and $k_{\mathrm{on}} = 0.05$ s^{-1} respectively.

As shown in Fig 2, both the vascular network dynamics (measured from parameters λ and $\delta_{\max}$) and the peak drug concentration in the tumour are fairly insensitive to the

polynomial order of function $m(\epsilon)$. Nonetheless, for the control simulation there is clear difference in the predicted tumour volume growth when $a_w = 2.0$, compared to the baseline results, because the peritumoural stromal tissue degradation (as an effect of the MMPs produced at the tumour front) is magnified in m quadratically.

Tumour tissue hydraulic conductivity

The baseline value for the tumour tissue hydraulic conductivity was set to $K_{\text{int-T}} = 2.5 \times 10^{-7}$ cm^2(mm-Hg s)$^{-1}$, while for the host tissue it was set to $K_{\text{int-H}} = 8.51 \times 10^{-9}$ cm^2(mm-Hg s)$^{-1}$ (see S1 Table). However, to test the effect of resistance to interstitial flow at the tumour (see Eq (2)) and its consequence to the delivery of the cytotoxic drugs, model parameter $K_{\text{int-T}}$ was allowed to take two extreme values with respect to the baseline: 2.5×10^{-8} cm^2(mm-Hg s)$^{-1}$ and 2.5×10^{-6} cm^2(mm-Hg s)$^{-1}$ respectively. It is important to highlight that this range falls within physiological values for the interstitium hydraulic conductivity of desmoplastic tumours, as reported for example from Netti and his colleagues [3]. As in the analysis of the previous paragraph, simulations were run for $r_p = 50$ nm poresize, and $k_{on} = 0.05$ s^{-1} drug affinity.

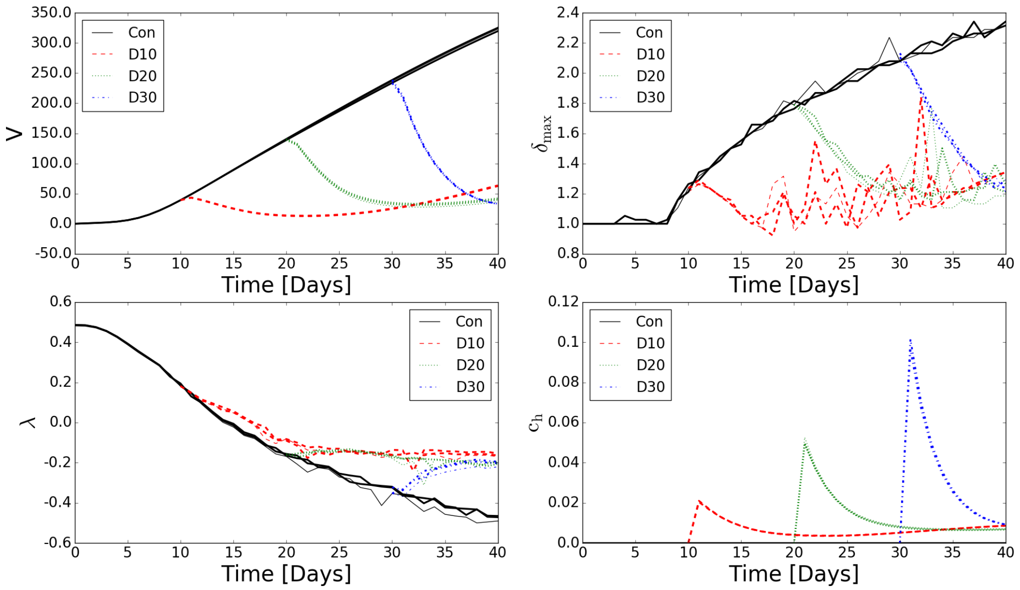

Fig 3. Tumour volume, vascular network structure and drug concentration as a result of tumour tissue hydraulic conductivity variations.

Line plots (from top to bottom and from left to right) of the relative tumour volume, V, the vascular network structural parameters δ_{max} and λ, and the drug concentrated in the tumour, c_h, as a function of time. Poresize and drug affinity were fixed at $r_p = 50$ nm and $k_{on} = 0.05$ s^{-1} respectively.

Fig 3 illustrates the time-development of the tumour volume, the vasculature's structural changes and the concentration of the drug inside the tumour, with both simulation results for the extreme values of $K_{\text{int-T}}$ superimposed to the baseline results reported in the manuscript. As can be seen from all line plots, the level of resistance to interstitial fluid flow neither affects the cancer mass development nor the extent of tumour regression due to the cytotoxic drug. The concentration of the latter also appears to be little affected by modifications of the $K_{\text{int-T}}$ model parameter. Finally,

the tumour vascular network structure is also not affected by changes to $K_{\text{int-T}}$ (some random noise in the line plots of δ_{max} for injection time day 10 is due to very rapid instantaneous changes of the vascular network remodelling and sprouting) and the perfusivity of the tumour vessels (data not shown) is also insensitive. The hydraulic conductivity of the tumour is related to fluid flow through the tumour interstitial space and thus, to the convective transport of drugs. In our study, we considered transport of chemotherapeutic agents, which are small in size (less than 1 nm) rendering transvascular and interstitial transport diffusion-dominated.

References

1. Baish JW, Stylianopoulos T, Lanning RM, Kamoun WS, Fukumura D, Munn LL, et al. Scaling rules for diffusive drug delivery in tumor and normal tissues. Proceedings of the National Academy of Sciences of the United States of America. 2011;108(5):1799–1803. doi:10.1073/pnas.1018154108.

2. Vavourakis V, Wijeratne PA, Shipley R, Loizidou M, Stylianopoulos T, Hawkes DJ. A Validated Multiscale In-Silico Model for Mechano-sensitive Tumour Angiogenesis and Growth. PLOS Computational Biology. 2017;13(1):e1005259. doi:10.1371/journal.pcbi.1005259.

3. Netti PA, Berk DA, Swartz MA, Grodzinsky AJ, Jain RK. Role of Extracellular Matrix Assembly in Interstitial Transport in Solid Tumors. Cancer Research. 2000;60(9):2497–2503.
