# Supplementary figures and images for: In-silico dynamic analysis of cytotoxic drug administration to solid tumours: Effect of binding affinity and vessel permeability
Source: PLoS Comput Biol. 2018 Oct 8;14(10):e1006460. doi: 10.1371/journal.pcbi.1006460 (PMC6193741; doi:10.1371/journal.pcbi.1006460)

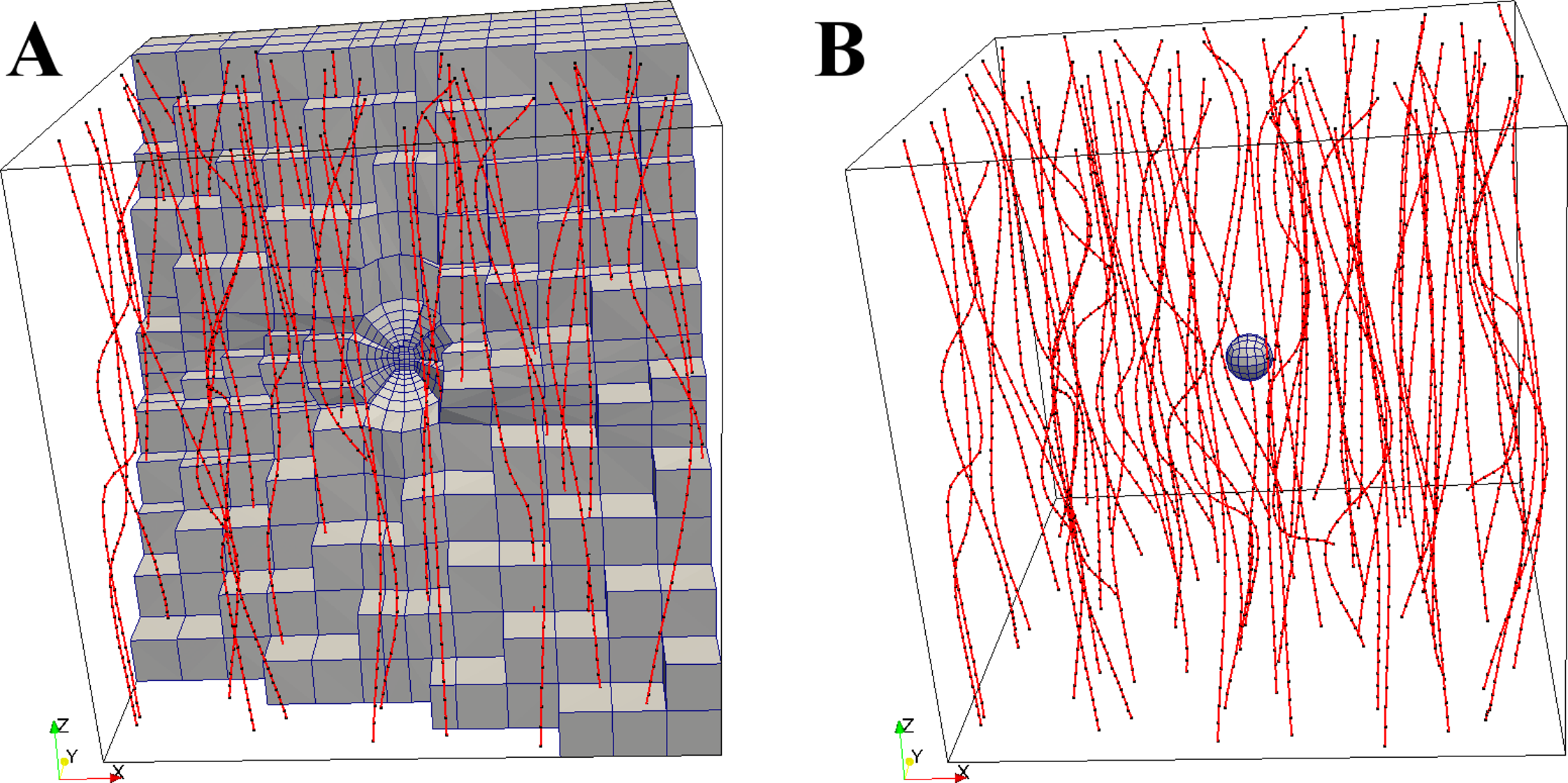

Supplement: S1 Fig — (A) Clipped mesh, showing the internal structure of the grid. (B) The extracted tumour region, shown here as a spheroid, with the complete vascular tree rendered as red segments and the black points denoting the vascular nodes. (TIF) [file pcbi.1006460.s003.tif]

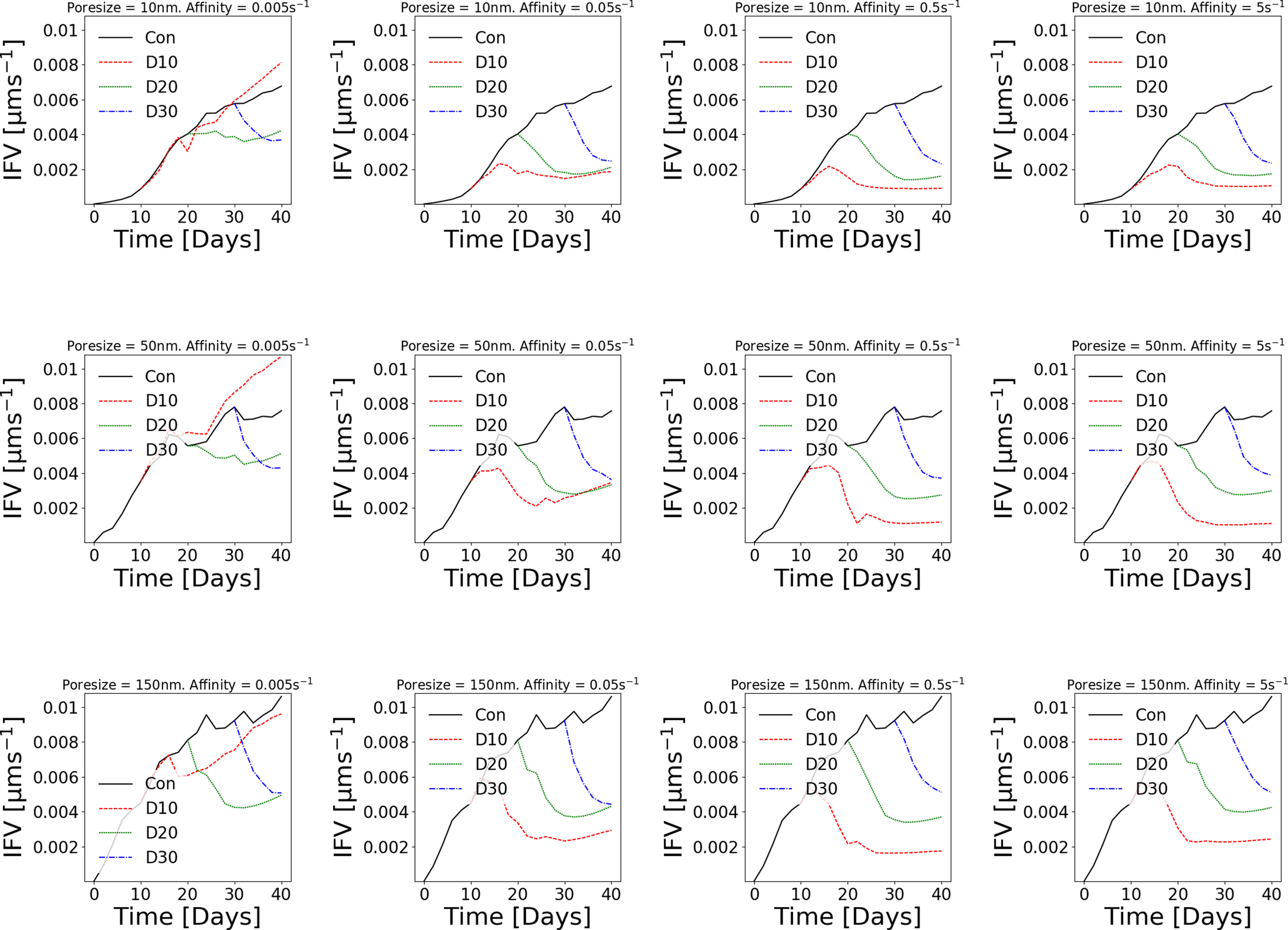

Supplement: S2 Fig — Line plots of the averaged IFV magnitude as a function of time. The 3×4 matrix of plots depicts the in-silico results (both the control and the treated cases) for three poresizes and four affinities: rp = 10 nm, 50 nm or 150 nm, and kon = 0.005 s-1, 0.05 s-1, 0.5 s-1 or 5 s-1, respectively. All sub-figures illustrate the predictions for the control and the treated case (drug injected at day 10 (D10), day 20 (D20) or day 30 (D30)). (TIF) [file pcbi.1006460.s004.tif]

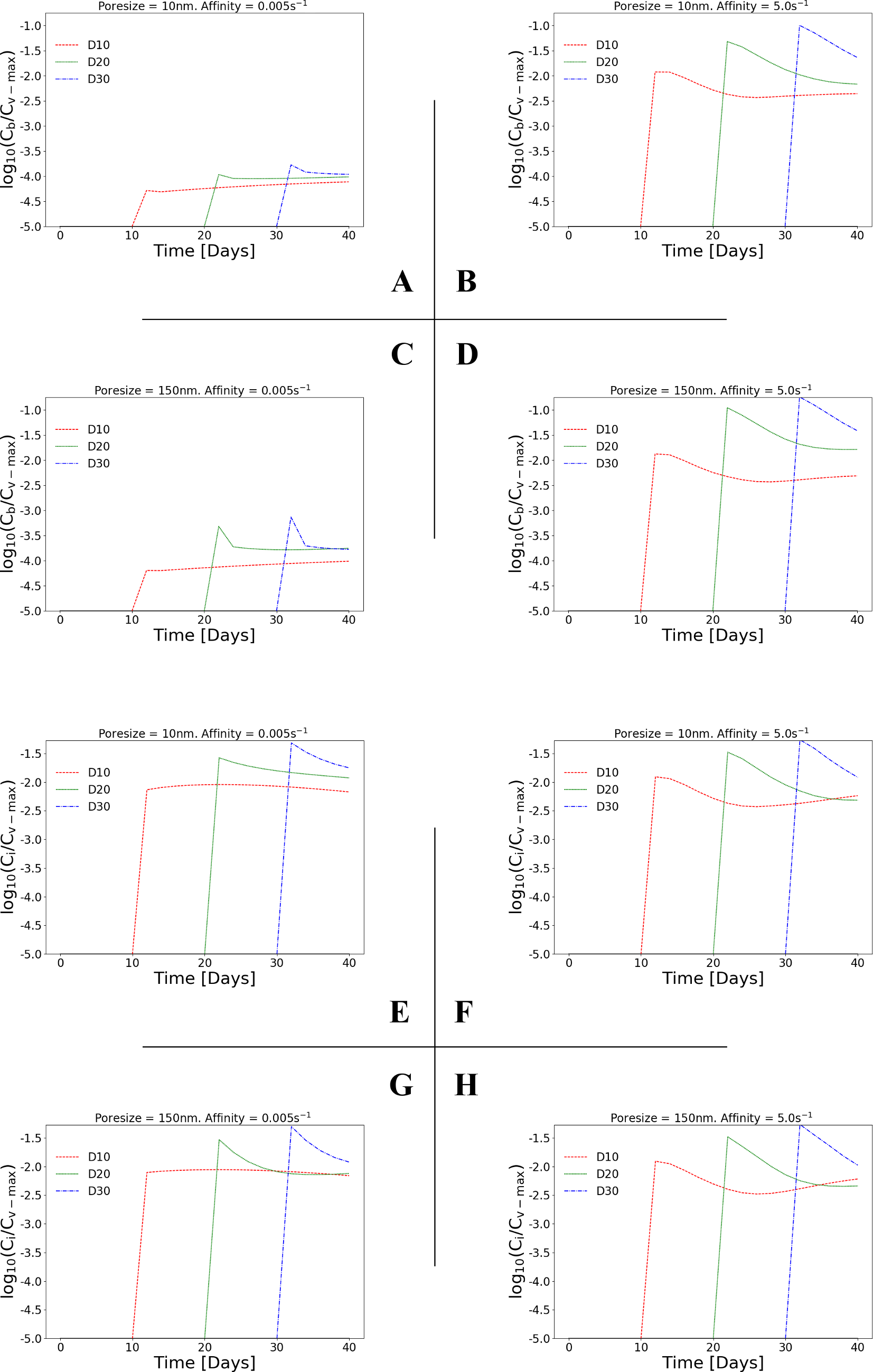

Supplement: S3 Fig — Line plots of the (A—D) bound/associated drug concentration, cb, and (E—H) internalised drug concentration, ci, expressed in dimensionless form (with respect to the injected drug concentration, cv-max) as a function of time. Each 2×2 matrix of plots depicts the in-silico results (treated case) for two poresizes: rp = 10 nm or 150 nm, and two affinities: kon = 0.005 s-1 or 5 s-1. (TIF) [file pcbi.1006460.s005.tif]

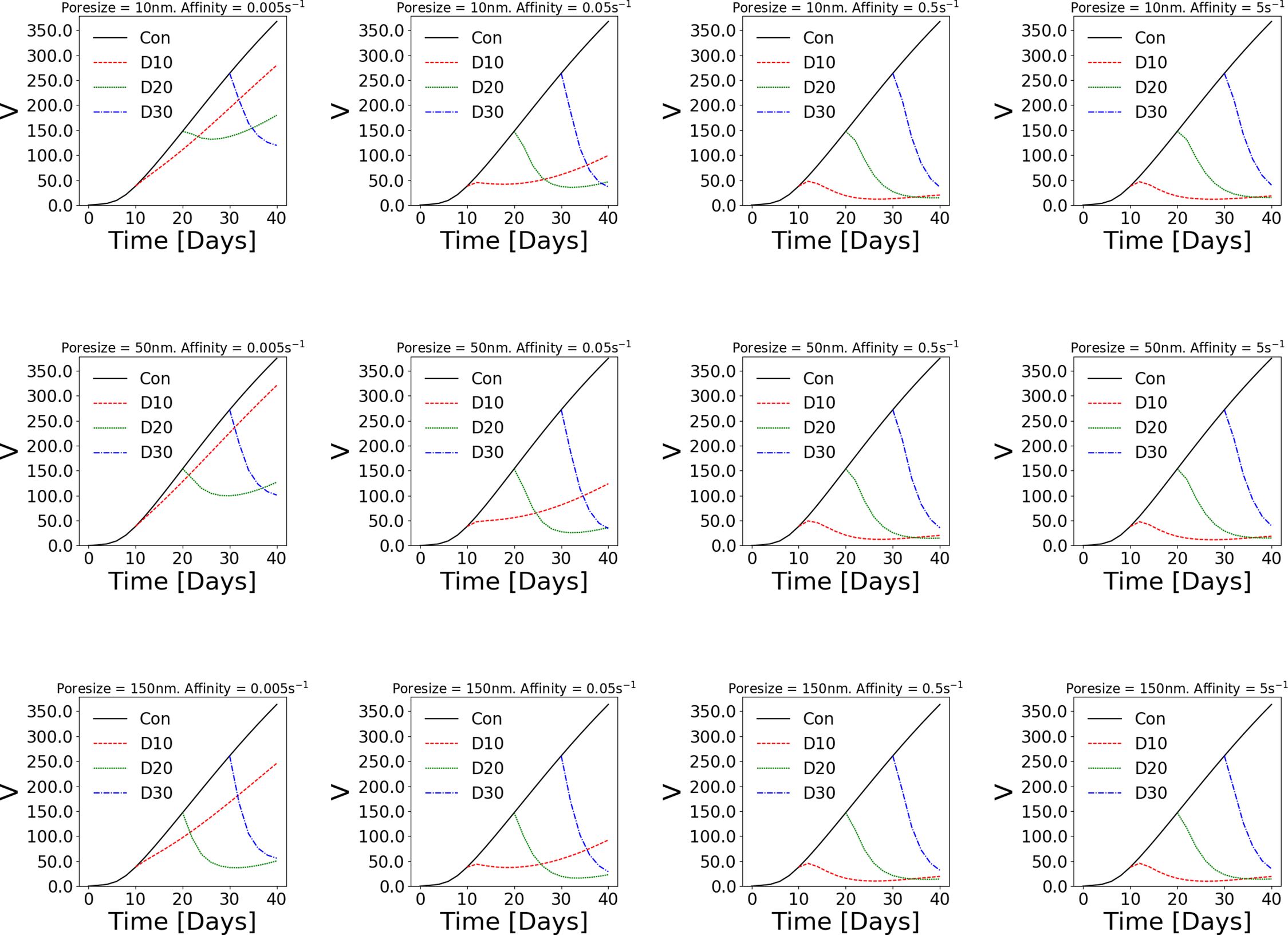

Supplement: S4 Fig — Line plots of the relative tumour volume (V = Vol.(t)/Vol.(t = 0)−1) as a function of time. The 3×4 matrix of plots depicts the in-silico results (both the control and the treated cases) for three poresizes and four affinities: rp = 10 nm, 50 nm or 150 nm, and kon = 0.005 s-1, 0.05 s-1, 0.5 s-1 or 5 s-1, respectively. All sub-figures illustrate the predictions for the control and the treated case (drug injected at day 10 (D10), day 20 (D20) or day 30 (D30)). (TIF) [file pcbi.1006460.s006.tif]

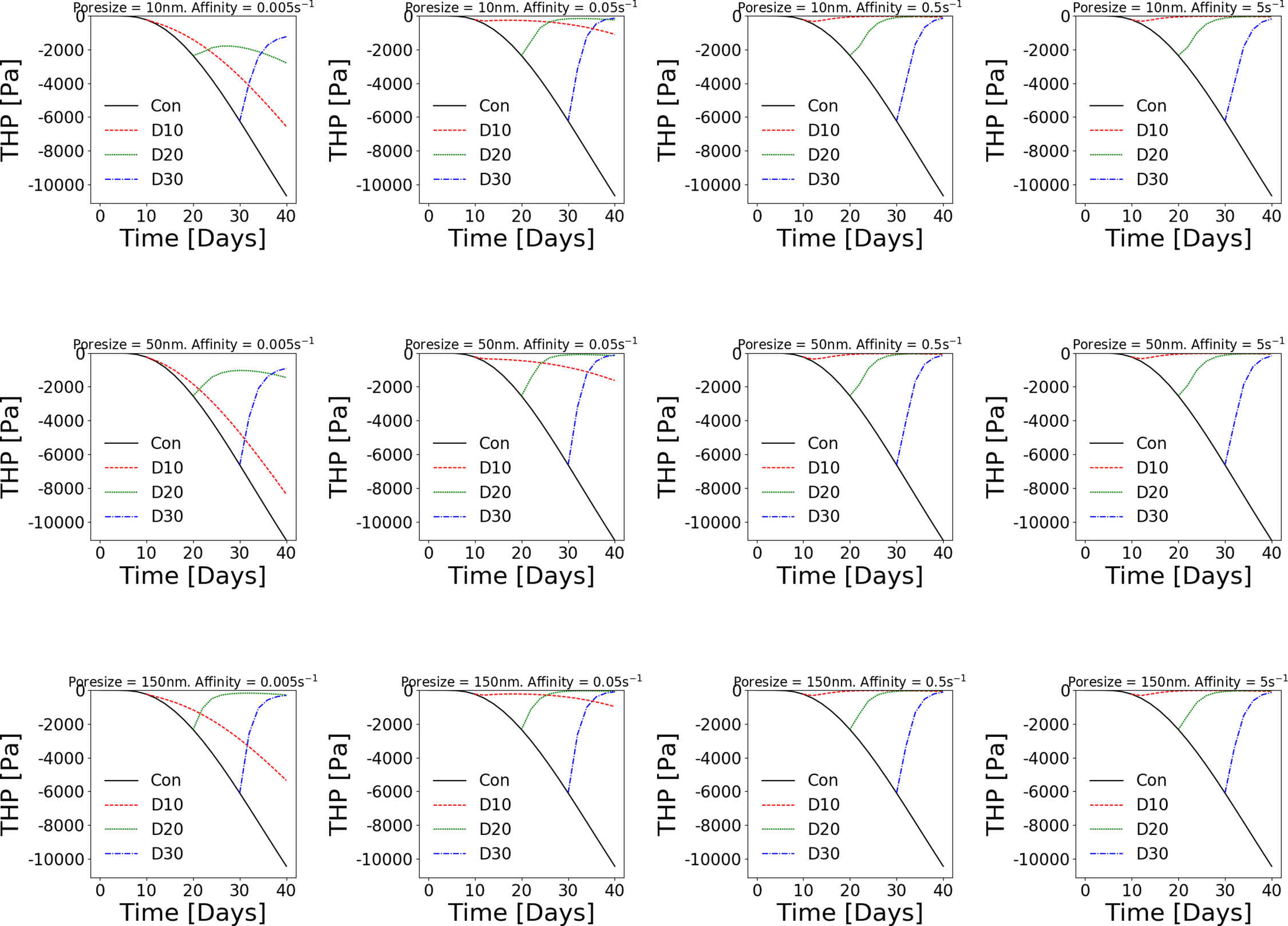

Supplement: S5 Fig — Line plots of THP as a function of time. The 3×4 matrix of plots depicts the in-silico results (both the control and the treated cases) for three poresizes and four affinities: rp = 10 nm, 50 nm or 150 nm, and kon = 0.005 s-1, 0.05 s-1, 0.5 s-1 or 5 s-1, respectively. All sub-figures illustrate the predictions for the control and the treated case (drug injected at day 10 (D10), day 20 (D20) or day 30 (D30)). (TIF) [file pcbi.1006460.s007.tif]

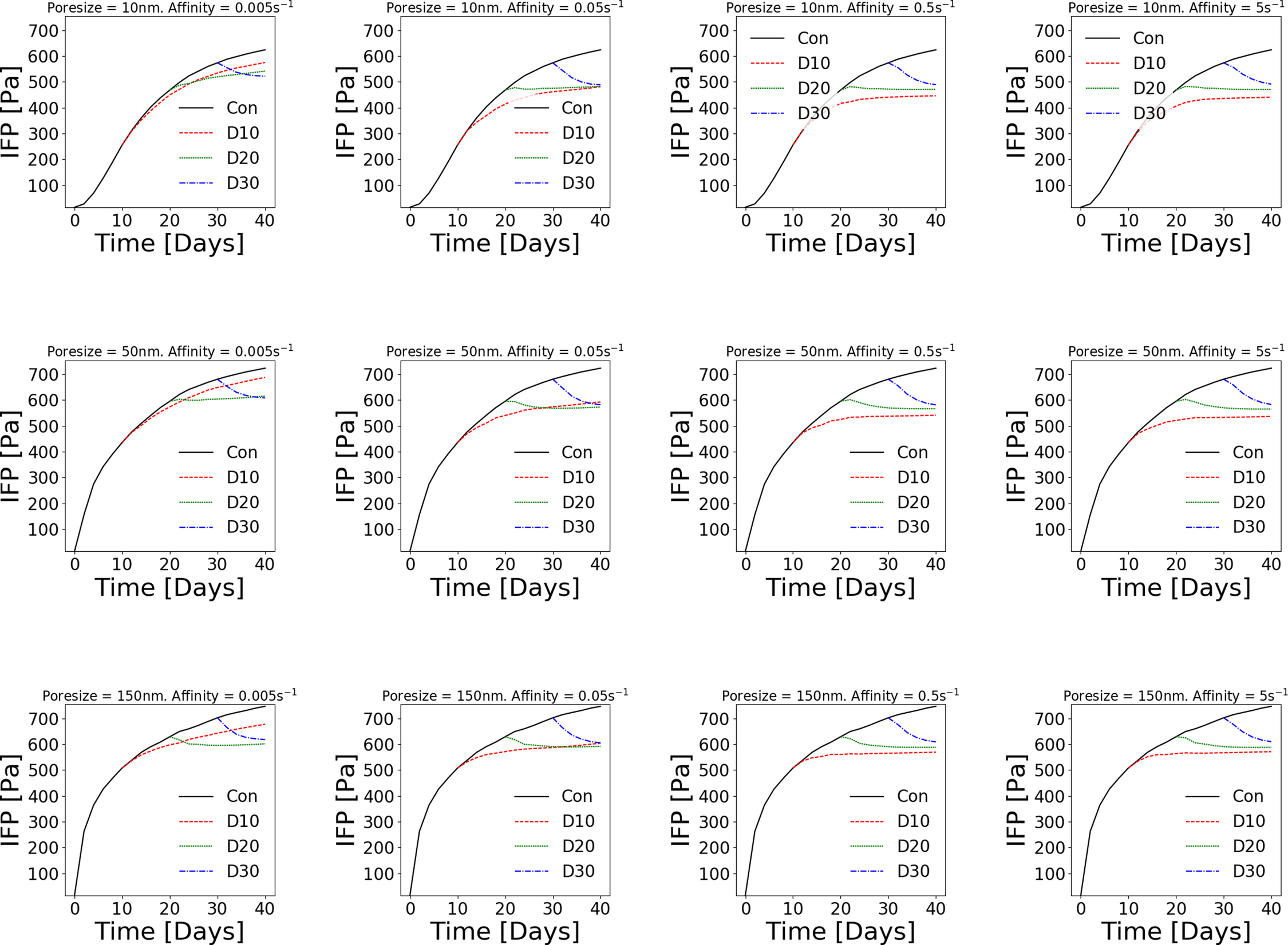

Supplement: S6 Fig — Line plots of IFP as a function of time. The 3×4 matrix of plots depicts the in-silico results (both the control and the treated cases) for three poresizes and four affinities: rp = 10 nm, 50 nm or 150 nm, and kon = 0.005 s-1, 0.05 s-1, 0.5 s-1 or 5 s-1, respectively. All sub-figures illustrate the predictions for the control and the treated case (drug injected at day 10 (D10), day 20 (D20) or day 30 (D30)). (TIF) [file pcbi.1006460.s008.tif]

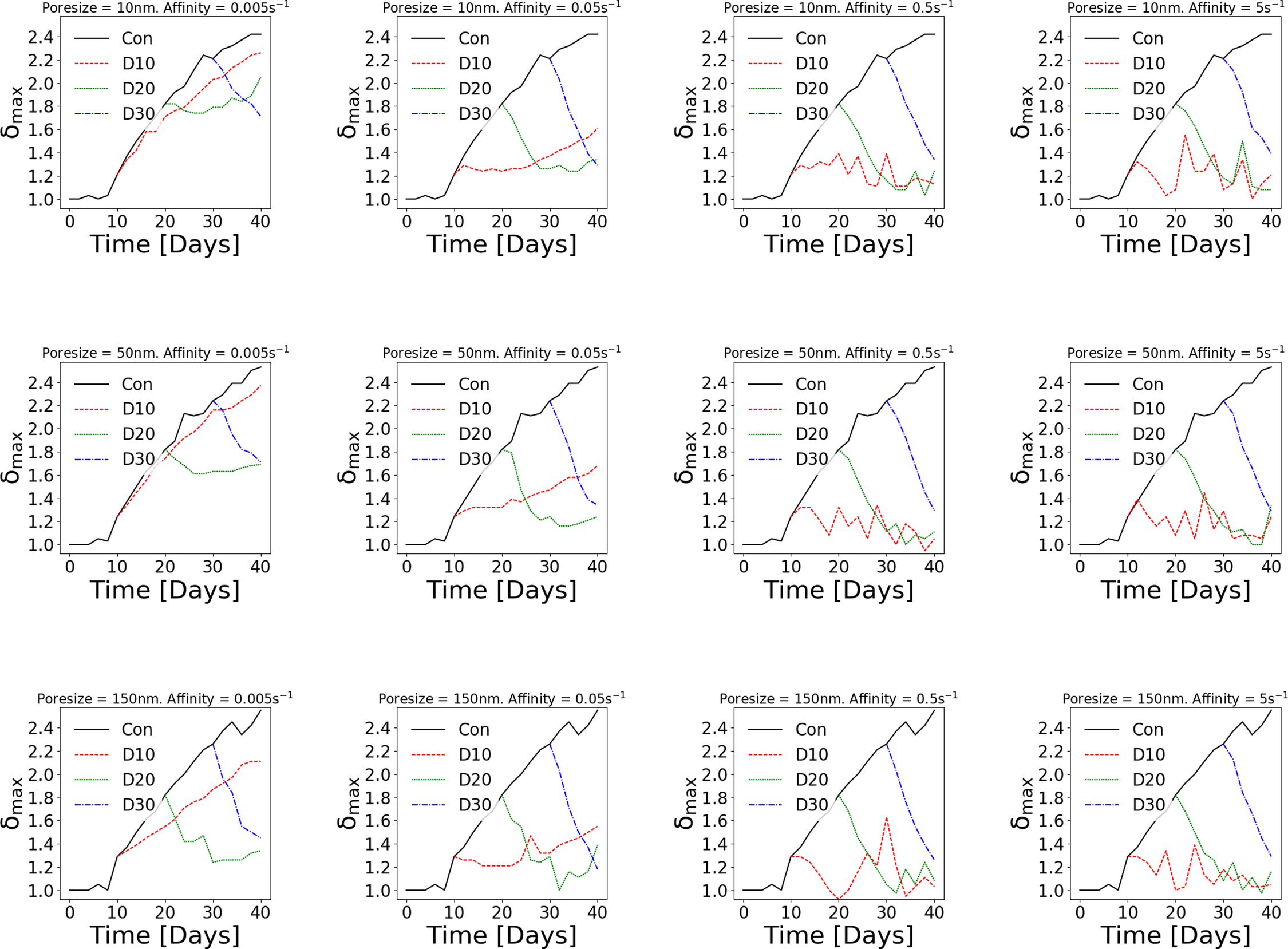

Supplement: S7 Fig — Line plots of normalised δmax as a function of time. The 3×4 matrix of plots depicts the in-silico results (both the control and the treated cases) for three poresizes and four affinities: rp = 10 nm, 50 nm or 150 nm, and kon = 0.005 s-1, 0.05 s-1, 0.5 s-1 or 5 s-1, respectively. All sub-figures illustrate the predictions for the control and the treated case (drug injected at day 10 (D10), day 20 (D20) or day 30 (D30)). (TIF) [file pcbi.1006460.s009.tif]

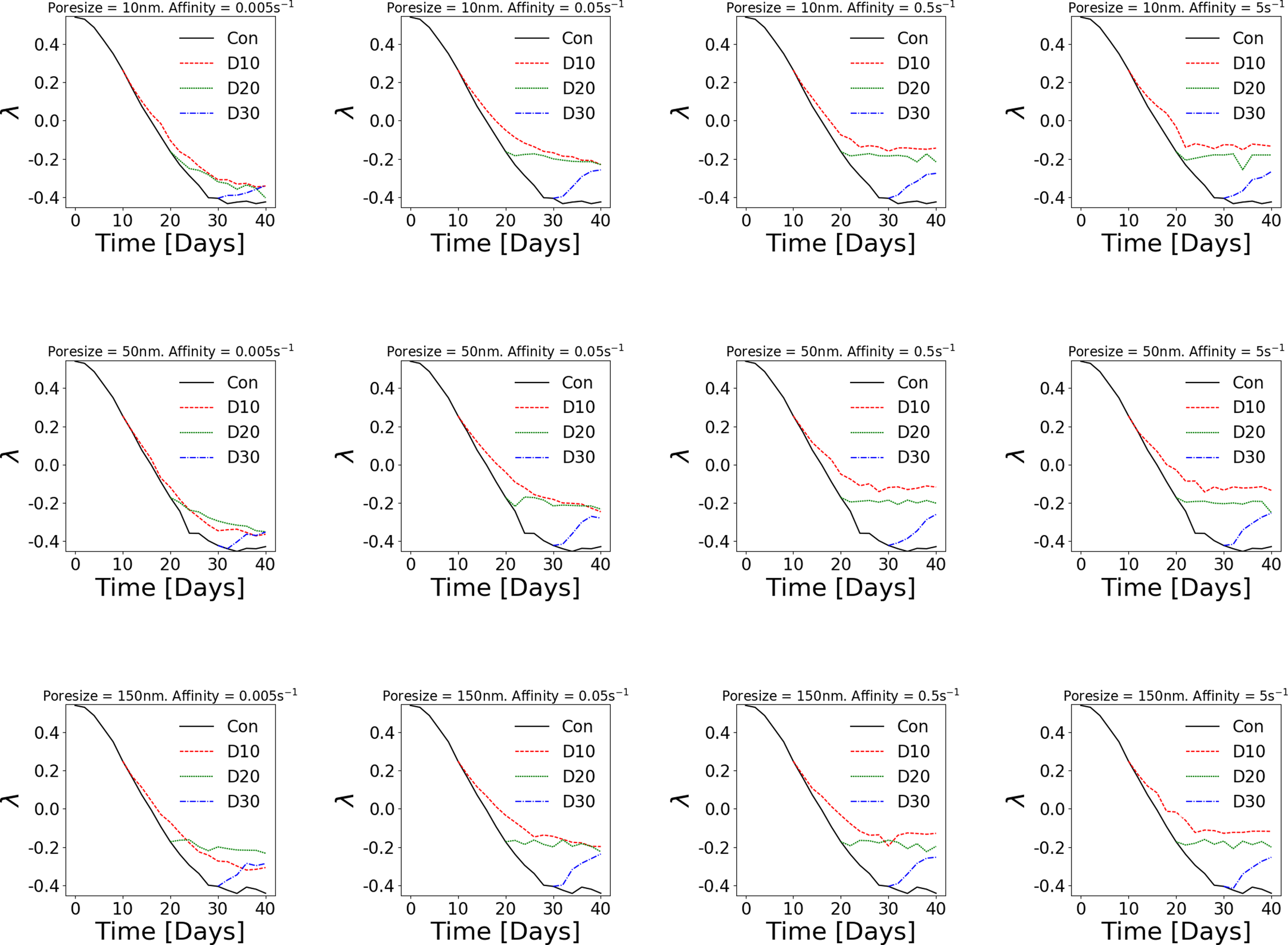

Supplement: S8 Fig — Line plots of λ as a function of time. The 3×4 matrix of plots depicts the in-silico results (both the control and the treated cases) for three poresizes and four affinities: rp = 10 nm, 50 nm or 150 nm, and kon = 0.005 s-1, 0.05 s-1, 0.5 s-1 or 5 s-1, respectively. All sub-figures illustrate the predictions for the control and the treated case (drug injected at day 10 (D10), day 20 (D20) or day 30 (D30)). (TIF) [file pcbi.1006460.s010.tif]

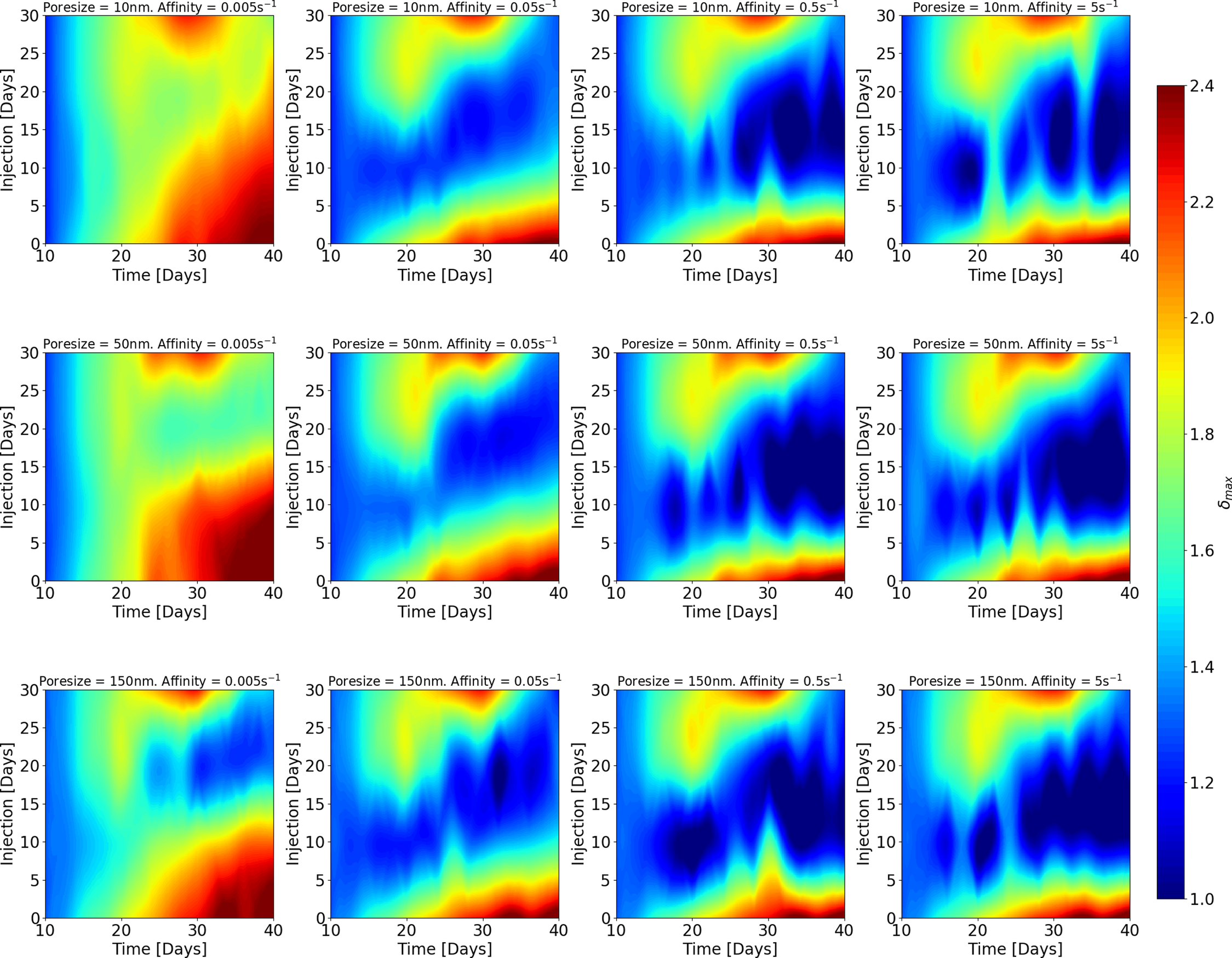

Supplement: S9 Fig — Contour plots of δmax as a function of time and injection time. The 3×4 matrix of plots depicts the in-silico results for three poresizes and four affinities: rp = 10 nm, 50 nm or 150 nm, and kon = 0.005 s-1, 0.05 s-1, 0.5 s-1 or 5 s-1, respectively. (TIF) [file pcbi.1006460.s011.tif]

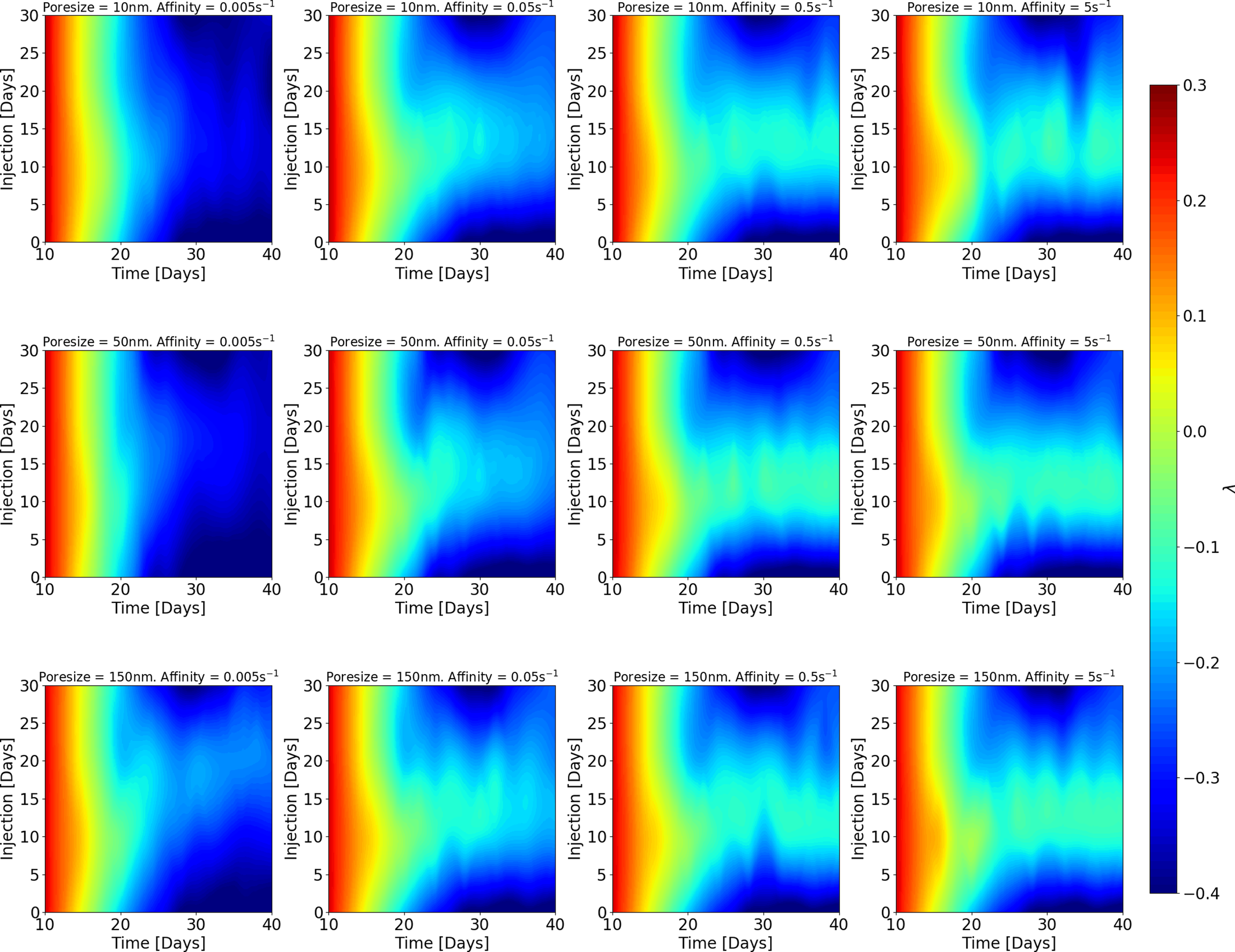

Supplement: S10 Fig — Contour plots of λ as a function of time and injection time. The 3×4 matrix of plots depicts the in-silico results for three poresizes and four affinities: rp = 10 nm, 50 nm or 150 nm, and kon = 0.005 s-1, 0.05 s-1, 0.5 s-1 or 5 s-1, respectively. (TIF) [file pcbi.1006460.s012.tif]

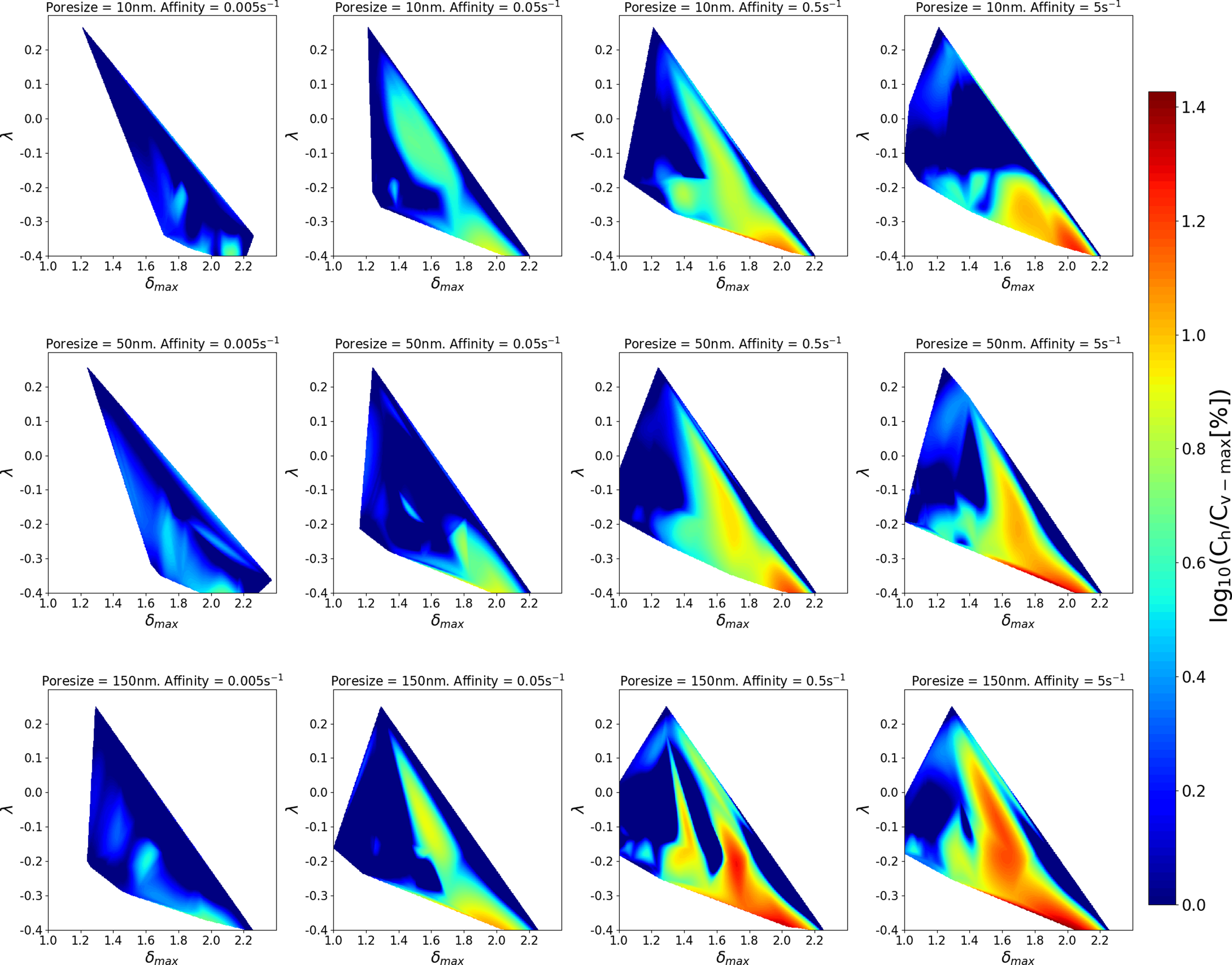

Supplement: S11 Fig — Contour plots of ch as a function of tumour development time and injection time. The 3×4 matrix of plots depicts the in-silico results for three poresizes and four affinities: rp = 10 nm, 50 nm or 150 nm, and kon = 0.005 s-1, 0.05 s-1, 0.5 s-1 or 5 s-1, respectively. (TIF) [file pcbi.1006460.s013.tif]

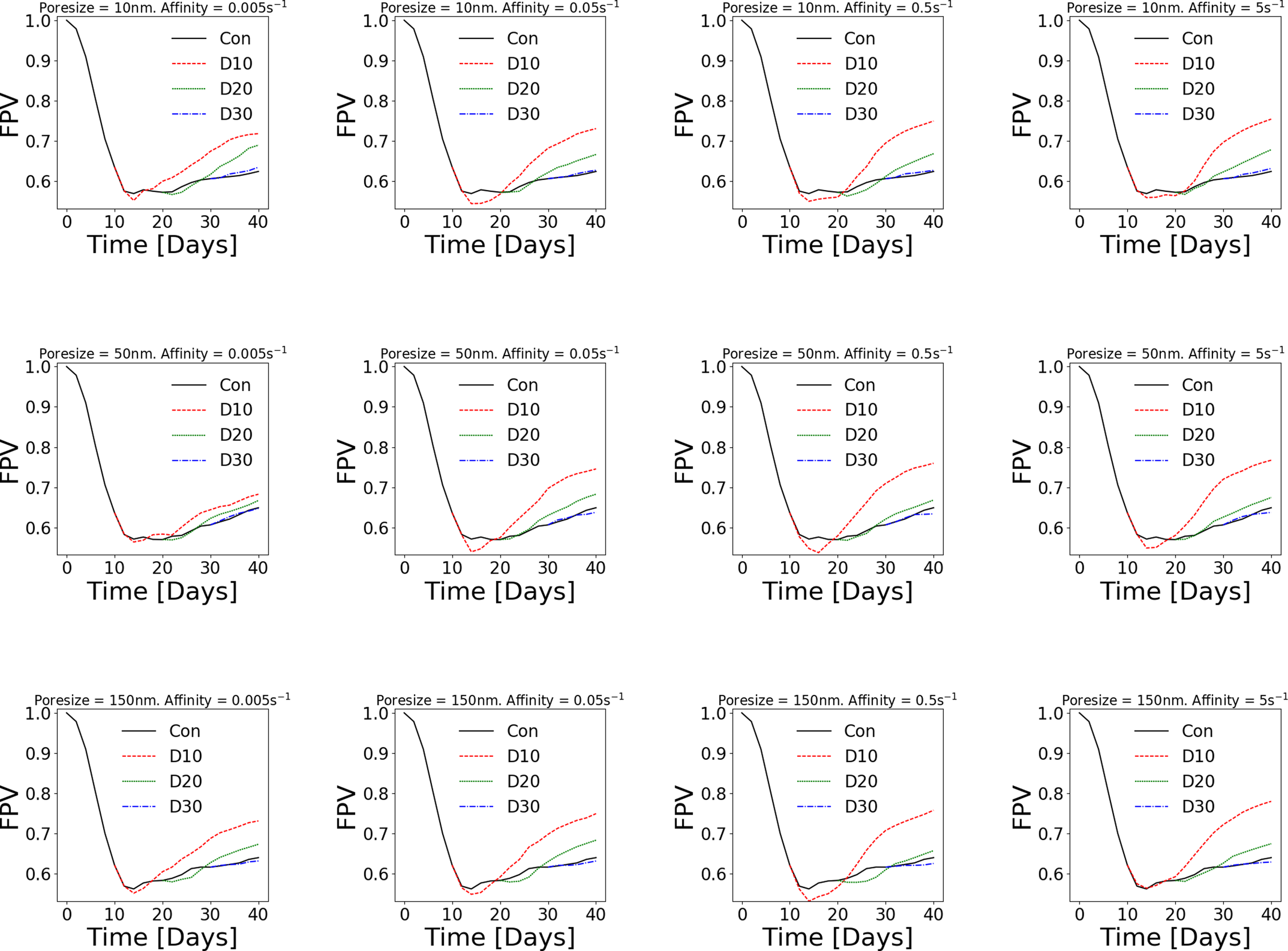

Supplement: S12 Fig — Line plots of the fraction of perfused vessels (FPV) as a function of time. FPV is described as the ratio of the length of all functional vessels that are sufficiently perfused (blood flow velocity is >0.1 mm s-1) to the length of all functional vessels (i.e. that have not collapsed). The 3×4 matrix of plots depicts the in-silico results (both the control and the treated cases) for three poresizes and four affinities: rp = 10 nm, 50 nm or 150 nm, and kon = 0.005 s-1, 0.05 s-1, 0.5 s-1 or 5 s-1, respectively. All sub-figures illustrate the predictions for the control and the treated case (drug injected at day 10 (D10), day 20 (D20) or day 30 (D30)). (TIF) [file pcbi.1006460.s014.tif]
