# Supplementary material for: In-silico dynamic analysis of cytotoxic drug administration to solid tumours: Effect of binding affinity and vessel permeability
Source: PLoS Comput Biol. 2018 Oct 8;14(10):e1006460. doi: 10.1371/journal.pcbi.1006460 (PMC6193741; doi:10.1371/journal.pcbi.1006460)
Supplement: S1 Table — List of model parameters associated with the Fluid Solver Module (see Fig 1). Cells marked with an asterisk denote shared values for both tissue types, while “VSC” denotes blood vessel and “LMP” denotes the lymphatic vessel. (PDF) [file pcbi.1006460.s021.pdf]

SUPPORTING INFORMATION

In-silico Dynamic Analysis of Cytotoxic Drug Administration to Solid Tumours: Effect of Binding Affinity and Vessel Permeability

Vasileios Vavourakis, Triantafyllos Stylianopoulos, Peter A. Wijeratne

Fluid mechanics model parameters

List of model parameters associated with the *Fluid Solver Module* (see Fig 1). Cells marked with an asterisk denote shared values for both tissue types, while "VSC" denotes blood vessel and "LMP" denotes the lymphatic vessel.

Parameter	Description	Host	Tumour	Source
[mm-Hg]	VSC Inlet: prescribed pressure	25.	*	[1]
[mm-Hg]	VSC Outlet: prescribed pressure	10.	*	[1]
μ_{B} [mm-Hg s]	blood viscosity	$3.e{-5}$	*	[2]
μ_{P} [mm-Hg s]	blood plasma viscosity	$1.125e{-5}$	*	[3]
μ_{I} [mm-Hg s]	interstitial fluid viscosity	$2.625e{-5}$	*	[3]
S_{vsc} [cm^{-1}]	VSC density	70.	200.	[4–6]
$K_{\text{int-}\{\mathrm{T}\|\mathrm{H}\}}$ [cm^2(mm-Hg s)$^{-1}$]	hydraulic conductivity of the interstitium	$8.51e{-9}$	$2.5e{-7}$	[7–9]
$K_{\mathrm{lmp}}\,S_{\mathrm{lmp}}$ [(mm-Hg s)$^{-1}$]	specific hydraulic permeability at LMP wall	$1.333e{-5}$	$1.333e{-9}$	this work
σ_{o} [–]	average osmotic reflection coefficient at VSC wall	0.91	$8.7e{-5}$	[10–12]
π_{vsc} [mm-Hg]	plasma osmotic pressure at VSC wall	20.	19.3	[12, 13]
π_{int} [mm-Hg]	interstitial fluid osmotic pressure	10.	17.3	[12, 14]
p_{int} [mm-Hg]	average IFP: initial value	0.1	*	[15]

References

1. Wu J, Xu S, Long Q, Collins MW, König CS, Zhao G, et al. Coupled modeling of blood perfusion in intravascular, interstitial spaces in tumor microvasculature. Journal of Biomechanics. 2008;41(5):996–1004. doi:10.1016/j.jbiomech.2007.12.008.

2. Fung YC. Biomechanics: Motion, Flow, Stress and Growth. New York, NY: Springer; 1990.

3. Chen CT, Malkus DS, Vanderby R. A fiber matrix model for interstitial fluid flow and permeability in ligaments and tendons. Biorheology. 1998;35(2):103–118. doi:10.1016/S0006-355X(99)80001-8.

4. Hilmas DE, Gillette EL. Morphometric analyses of the microvasculature of tumors during growth and after X-irradiation. Cancer. 1974;33(1):103–110.

5. Jain RK. Transport of molecules across tumor vasculature. Cancer Metastasis Rev. 1987;6:559–594.

6. Soltani M, Chen P. Numerical Modeling of Fluid Flow in Solid Tumors. PLoS ONE. 2011;6(6):e20344. doi:10.1371/journal.pone.0020344.

7. Swabb EA, Wei J, Gullino PM. Diffusion and Convection in Normal and Neoplastic Tissues. Cancer Research. 1974;34(10):2814–2822.

8. Jain RK. Transport of molecules in the tumor interstitium: A review. Cancer Research. 1987;47:3039–3051.

9. Netti PA, Berk DA, Swartz MA, Grodzinsky AJ, Jain RK. Role of Extracellular Matrix Assembly in Interstitial Transport in Solid Tumors. Cancer Research. 2000;60(9):2497–2503.

10. Ballard K, Perl W. Osmotic reflection coefficients of canine subcutaneous adipose tissue endothelium. Microvascular Research. 1978;16(2):224–236.

11. Curry FE. Handbook of Physiology, Section 2: The Cardiovascular System. Bethesda, MD: American Physiological Society; 1984. p. 309–374.

12. Jain RK, Tong RT, Munn LL. Effect of vascular normalization by antiangiogenic therapy on interstitial hypertension, peritumor edema, and lymphatic metastasis: Insights from a mathematical model. Cancer Research. 2007;67(6):2729–2735. doi:10.1158/0008-5472.CAN-06-4102.

13. Brace RA, Guyton AC. Interaction of transcapillary Starling forces in the isolated dog forelimb. American Journal of Physiology - Heart and Circulatory Physiology. 1977;233(1):H136–H140.

14. Baxter LT, Jain RK. Transport of fluid and macromolecules in tumors. I. Role of interstitial pressure and convection. Microvascular Research. 1989;37(1):77–104. doi:10.1016/0026-2862(89)90074-5.

15. Vavourakis V, Wijeratne PA, Shipley R, Loizidou M, Stylianopoulos T, Hawkes DJ. A Validated Multiscale In-Silico Model for Mechano-sensitive Tumour Angiogenesis and Growth. PLOS Computational Biology. 2017;13(1):e1005259. doi:10.1371/journal.pcbi.1005259.
