# Supplementary material for: In-silico dynamic analysis of cytotoxic drug administration to solid tumours: Effect of binding affinity and vessel permeability
Source: PLoS Comput Biol. 2018 Oct 8;14(10):e1006460. doi: 10.1371/journal.pcbi.1006460 (PMC6193741; doi:10.1371/journal.pcbi.1006460)
Supplement: S2 Table — List of model parameters associated with the Vascular Network Module (see Fig 1). Parameters with a star (⋆) correspond to non-perfused or hypo-perfused vessels, while those with a dagger (†) correspond to well-perfused vessels. The parameters with a double dagger (‡) denote the pre-set parameter values of the original vascular network, while the cell marked with an asterisk denotes shared value for both tissue types. (PDF) [file pcbi.1006460.s022.pdf]

SUPPORTING INFORMATION

In-silico Dynamic Analysis of Cytotoxic Drug Administration to Solid Tumours: Effect of Binding Affinity and Vessel Permeability

Vasileios Vavourakis, Triantafyllos Stylianopoulos, Peter A. Wijeratne

Vascular network model parameters

List of model parameters associated with the *Vascular Network Module* (see Fig 1). Parameters with a star ($\star$) correspond to non-perfused or hypo-perfused vessels, while those with a dagger (†) correspond to well-perfused vessels. The parameters with a double dagger (‡) denote the pre-set parameter values of the original vascular network, while the cell marked with an asterisk denotes shared value for both tissue types.

**Parameter**	**Description**	**Host**	**Tumour**	**Source**
k_τ [m]	chemotaxis in angiogenesis	1.		[1]
k_ϵ [m]	haptotaxis in angiogenesis	0.3		[1]
k_{m} [Pa^{-1}]	mechanotaxis in angiogenesis	0.01		[1]
$\tilde{R}$ [µm]	see Eq (16) in [1]	4.		[2]
$v_{\text{v-0}}$, $v_{\text{v-1}}$ [µm d^{-1}]	see Eq (16) in [1]	5.5, 479.7		[2]
$v_{\text{v-max}}$ [µm d^{-1}]		250.		[1, 2]
τ^* [–]	TAF threshold above which angiogenesis occurs	0.01		[1]
μ_{D}, σ_{D} [µm]	median and standard deviation of distance-related probability function $\mathcal{P}_{\mathrm{D}}$	$150. \pm 15^\star$, $300. \pm 30^\dagger$		[1]
μ_{A}, σ_{A} [d]	median and standard deviation of age-related probability function $\mathcal{P}_{\mathrm{A}}$	$0.5 \pm 0.25^\star$, $1.25 \pm 0.25^\dagger$		[1]
[µm]	minimum distance (between two different branches) to enforce anastomosis	40.		[1]
R [µm]	capillary lumen radius of original (host) vessels; range of R for tumour vessels	$80.^\ddagger$; 5.—60.		[3, 4]
h [µm]	capillary wall thickness of original vessels; range of h for tumour vessels	$5.5^\ddagger$; 3.—5.		[3, 4]
r_{p} [nm]	capillary wall pore size of original vessels; average value of r_{p} for tumour vessels	$1.^\ddagger$; [10., 50., 150.]		[5, 6]
γ_{p} [–]	fraction of endothelium surface occupied by pores for original vessels; value of γ_{p} for tumour vessels	$1.e{-}4^\ddagger$; $10.e{-}4$		[7]

References

1. Vavourakis V, Wijeratne PA, Shipley R, Loizidou M, Stylianopoulos T, Hawkes DJ. A Validated Multiscale In-Silico Model for Mechano-sensitive Tumour Angiogenesis and Growth. PLOS Computational Biology. 2017;13(1):e1005259. doi:10.1371/journal.pcbi.1005259.

2. Wood LB, Ge R, Kamm RD, Asada HH. Nascent vessel elongation rate is inversely related to diameter in in vitro angiogenesis. Integrative Biology. 2012;4:3579–3600.

3. Hashizume H, Baluk P, Morikawa S, McLean JW, Thurston G, Roberge S, et al. Openings between Defective Endothelial Cells Explain Tumor Vessel Leakiness. The American Journal of Pathology. 2000;156(4):1363–1380. doi:10.1016/S0002-9440(10)65006-7.

4. Morikawa S, Baluk P, Kaidoh T, Haskell A, Jain RK, McDonald DM. Abnormalities in Pericytes on Blood Vessels and Endothelial Sprouts in Tumors. The American Journal of Pathology. 2002;160(3):985–1000. doi:10.1016/S0002-9440(10)64920-6.

5. Sarin H. Physiologic upper limits of pore size of different blood capillary types and another perspective on the dual pore theory of microvascular permeability. Journal of Angiogenesis Research. 2010;2(1):1–19. doi:10.1186/2040-2384-2-14.

6. Chauhan VP, Stylianopoulos T, Martin JD, Popovic Z, Chen O, Kamoun WS, et al. Normalization of tumour blood vessels improves the delivery of nanomedicines in a size-dependent manner. Nature Nanotechnology. 2012;7(6):383–388. doi:10.1038/nnano.2012.45.

7. Jain RK, Tong RT, Munn LL. Effect of vascular normalization by antiangiogenic therapy on interstitial hypertension, peritumor edema, and lymphatic metastasis: Insights from a mathematical model. Cancer Research. 2007;67(6):2729–2735. doi:10.1158/0008-5472.CAN-06-4102.
