# Supplementary material for: In-silico dynamic analysis of cytotoxic drug administration to solid tumours: Effect of binding affinity and vessel permeability
Source: PLoS Comput Biol. 2018 Oct 8;14(10):e1006460. doi: 10.1371/journal.pcbi.1006460 (PMC6193741; doi:10.1371/journal.pcbi.1006460)
Supplement: S3 Table — List of model parameters associated with the Biochemical Solver Module and the Drug Delivery Solver Module (see Fig 1). Cells marked with an asterisk denote shared values for both tissue types, while “NA” denotes non-applicable. (PDF) [file pcbi.1006460.s023.pdf]

SUPPORTING INFORMATION

In-silico Dynamic Analysis of Cytotoxic Drug Administration to Solid Tumours: Effect of Binding Affinity and Vessel Permeability

Vasileios Vavourakis, Triantafyllos Stylianopoulos, Peter A. Wijeratne

Biochemical and drug delivery model parameters

List of model parameters associated with the *Biochemical Solver Module* and the *Drug Delivery Solver Module* (see Fig 1). Cells marked with an asterisk denote shared values for both tissue types, while "NA" denotes non-applicable.

**Parameter**	**Description**	**Host**	**Tumour**	**Source**
λ_ϵ [d^{-1}]	ECM remodelling rate	$8.64e{-4}$	$8.64e{-1}$	adapted from [1]
δ_ϵ [d^{-1}]	ECM degradation rate from MDEs	7.465	NA	[2]
δ_d [d^{-1}]	ECM degradation rate from drug	0.	$8.64e{-2}$	this work
$\bar{\epsilon}$ [–]	ECM remodelling threshold	0.33	*	[2]
$\bar{\epsilon}_\mathrm{d}$ [–]	ECM depletion threshold (due to drug), see Eq (13)	NA	0.1	this work
$\bar{c}_\mathrm{i}$ [–]	see Eq (13)	NA	0.01	this work
a_d [–]	see Eq (13)	NA	2.5	this work
s_c [nm]	size of drug molecule, see Eq (9)	1.		
τ_c [h]	half-life of the drug	4.		this work
k_on [s^{-1}]	drug association (affinity) rate	0.	0.005, 0.05, 0.5, 5.	adapted from [3]
k_off [s^{-1}]	drug disassociation rate	0.	$1.e{-4}$	[3]
k_int [s^{-1}]	drug internalisation rate	0.	$5.e{-5}$	[3]
δ_b [s^{-1}]	internalised drug decay rate	0.	$0.0864e{-5}$	this work

References

1. Wood LB, Ge R, Kamm RD, Asada HH. Nascent vessel elongation rate is inversely related to diameter in in vitro angiogenesis. Integrative Biology. 2012;4:3579–3600.

2. Vavourakis V, Wijeratne PA, Shipley R, Loizidou M, Stylianopoulos T, Hawkes DJ. A Validated Multiscale In-Silico Model for Mechano-sensitive Tumour Angiogenesis and Growth. PLOS Computational Biology. 2017;13(1):e1005259. doi:10.1371/journal.pcbi.1005259.

3. Stylianopoulos T, Jain RK. Combining two strategies to improve perfusion and drug delivery in solid tumors. Proceedings of the National Academy of Sciences of the United States of America. 2013;110(46):18632–18637. doi:10.1073/pnas.1318415110.
