# Supplementary material for: In-silico dynamic analysis of cytotoxic drug administration to solid tumours: Effect of binding affinity and vessel permeability
Source: PLoS Comput Biol. 2018 Oct 8;14(10):e1006460. doi: 10.1371/journal.pcbi.1006460 (PMC6193741; doi:10.1371/journal.pcbi.1006460)
Supplement: S4 Table — List of model parameters associated with the Solid Solver Module (see Fig 1). Cells marked with an asterisk denote shared values for both tissue types, while “NA” denotes non-applicable. (PDF) [file pcbi.1006460.s024.pdf]

SUPPORTING INFORMATION

In-silico Dynamic Analysis of Cytotoxic Drug Administration to Solid Tumours: Effect of Binding Affinity and Vessel Permeability

Vasileios Vavourakis, Triantafyllos Stylianopoulos, Peter A. Wijeratne

Solid mechanics model parameters

List of model parameters associated with the *Solid Solver Module* (see Fig 1). Cells marked with an asterisk denote shared values for both tissue types, while "NA" denotes non-applicable.

Parameter	Description	Host	Tumour	Source
μ, κ [kPa]	Mooney-Rivlin model	10., 23.3	37.8, 50.4	[1–3]
α_g, β_g, γ_g, δ_g [−]	isotropic growth parameters	NA	3., 12.8, 6., 3.	[3]
a_w [−]	structural integrity exponent	NA	1.	this work

References

1. Voutouri C, Mpekris F, Papageorgis P, Odysseos AD, Stylianopoulos T. Role of Constitutive Behavior and Tumor-Host Mechanical Interactions in the State of Stress and Growth of Solid Tumors. PLoS ONE. 2014;9(8):e104717. doi:10.1371/journal.pone.0104717.

2. Wijeratne PA, Vavourakis V, Hipwell JH, Voutouri C, Papageorgis P, Stylianopoulos T, et al. Multiscale modelling of solid tumour growth: the effect of collagen micromechanics. Biomechanics and Modeling in Mechanobiology. 2015; p. 1–12. doi:10.1007/s10237-015-0745-2.

3. Vavourakis V, Wijeratne PA, Shipley R, Loizidou M, Stylianopoulos T, Hawkes DJ. A Validated Multiscale In-Silico Model for Mechano-sensitive Tumour Angiogenesis and Growth. PLOS Computational Biology. 2017;13(1):e1005259. doi:10.1371/journal.pcbi.1005259.
